# An Evolutionary Computation Approach to Examine Functional Brain Plasticity

**DOI:** 10.3389/fnins.2016.00146

**Published:** 2016-04-05

**Authors:** Arnab Roy, Colin Campbell, Rachel A. Bernier, Frank G. Hillary

**Affiliations:** ^1^Department of Psychology, The Pennsylvania State UniversityUniversity Park, PA, USA; ^2^Social Life and Engineering Imaging Center, The Pennsylvania State UniversityUniversity Park, PA, USA; ^3^Department of Physics, Washington CollegeChestertown, MD, USA; ^4^Department of Neurology, Hershey Medical CenterHershey, PA, USA

**Keywords:** fMRI, evolutionary computation, network plasticity, voxel-based approach, traumatic brain injury

## Abstract

One common research goal in systems neurosciences is to understand how the functional relationship between a pair of regions of interest (ROIs) evolves over time. Examining neural connectivity in this way is well-suited for the study of developmental processes, learning, and even in recovery or treatment designs in response to injury. For most fMRI based studies, the strength of the functional relationship between two ROIs is defined as the correlation between the average signal representing each region. The drawback to this approach is that much information is lost due to averaging heterogeneous voxels, and therefore, the functional relationship between a ROI-pair that evolve at a spatial scale much finer than the ROIs remain undetected. To address this shortcoming, we introduce a novel evolutionary computation (EC) based voxel-level procedure to examine functional plasticity between an investigator defined ROI-pair by simultaneously using subject-specific BOLD-fMRI data collected from two sessions seperated by finite duration of time. This data-driven procedure detects a sub-region composed of spatially connected voxels from each ROI (a so-called sub-regional-pair) such that the pair shows a significant gain/loss of functional relationship strength across the two time points. The procedure is recursive and iteratively finds all statistically significant sub-regional-pairs within the ROIs. Using this approach, we examine functional plasticity between the default mode network (DMN) and the executive control network (ECN) during recovery from traumatic brain injury (TBI); the study includes 14 TBI and 12 healthy control subjects. We demonstrate that the EC based procedure is able to detect functional plasticity where a traditional averaging based approach fails. The subject-specific plasticity estimates obtained using the EC-procedure are highly consistent across multiple runs. Group-level analyses using these plasticity estimates showed an increase in the strength of functional relationship between DMN and ECN for TBI subjects, which is consistent with prior findings in the TBI-literature. The EC-approach also allowed us to separate sub-regional-pairs contributing to positive and negative plasticity; the detected sub-regional-pairs significantly overlap across runs thus highlighting the reliability of the EC-approach. These sub-regional-pairs may be useful in performing nuanced analyses of brain-behavior relationships during recovery from TBI.

## Introduction

The human brain is an ever-changing network of complex interactions between billions of neurons operating at multiple spatio-temporal scales (Hebb, 1947; Diamond et al., 1981; Greenough and Chang, 1988). Recent applications of blood oxygen level dependent (BOLD) functional magnetic resonance imaging (fMRI) have focused on examining the functional relationship between spatially discrete regions at a macroscopic level (Biswal et al., 1995; Raichle et al., 2001; Fox et al., 2005; Greicius et al., 2009). In fMRI literature, the functional relationship between a pair of regions of interest (ROIs) in the brain has been traditionally defined as the degree of temporal synchronization between the regions (Friston et al., 1993). A common research goal in the systems neuroscience is to examine changes in brain connectivity over time. For example, in a study examining the influence of exercise in cognitive aging, investigators examined the connectivity changes between two predefined temporal regions, bilateral parahippocampus, and bilateral middle temporal gyrus (Voss et al., 2013). Alternatively, in the context of learning, changes in the functional relationship between areas associated with motor and sensory cortices, such as A1 and the insula and M1 and thalamus, have been examined following musical training (Luo et al., 2012). The clinical neurosciences have a burgeoning literature examining the consequences of neurological insult and normal and abnormal aging on large-scale brain networks (for review of the literatures see Sheline and Raichle, 2013; Tijms et al., 2013; Hillary et al., 2015). For example, in traumatic brain injury (TBI), there has been increasing interest in documenting the change in pairwise functional relationships among the regions central to large scale networks, such as the default mode network (DMN), salience network, and the executive control network (ECN; Hillary et al., 2011a, 2014; Sharp et al., 2011; Bonnelle et al., 2012; Arenivas et al., 2014; Venkatesan et al., 2014). It is the primary aim in these literatures to understand how the functional relationship between predefined regions in the brain evolves over time.

Common approaches to examine functional brain connectivity include seed-based or anatomical-atlas (van den Heuvel and Hulshoff Pol, 2010) based analysis. In the former, the relationship of a targeted brain-region (a seed) is correlated with all other brain regions in a pairwise manner. Alternatively, examiners may use an atlas-based approach and ROIs are defined using standardized anatomical atlases (e.g., Automated Anatomical Labeling; Tzourio-Mazoyer et al., 2002) and the functional relationship between any pair of ROIs is defined as the correlation between the average signal representing each ROI (Luo et al., 2012; Hillary et al., 2014; Rajtmajer et al., 2015). In an ideal scenario, a ROI would be composed of voxels that represent homogeneous activity. However, the reliance upon structural (as opposed to functional) information (e.g., atlas-based approach) results in ROIs with significant signal heterogeneity. Within ROI heterogeneity may cause the functional relationship between two ROIs to change across time at finer spatial scales than the ROIs themselves, and these changes may not be easily detected using an averaging based approach. To minimize within ROI heterogeneity, a number of data-driven voxel-level methods have been developed to aid in ROI definition (Heller et al., 2006; Yan et al., 2011; Zalesky et al., 2012; Thirion et al., 2014; Yeo et al., 2014; Rajtmajer et al., 2015; Wang et al., 2015). However, traditional ROI parcellation approaches are not designed to detect functional connectivity changes between two ROIs that are occurring at finer spatial scales. Hence there is a need for a data driven approach that can automatically detect the correct spatial scales at which the functional connectivity between 2 ROIs are changing across time.

In order to better examine the fine scale functional connectivity changes between two brain regions across time all voxel-wise interactions between the regional-pairs must be examined. One way to handle this is by defining the strength of the functional relationship between a regional-pair as the number of statistically significant voxel-wise functional connections that exist between the two regions (where two voxels are functionally connected if the activity at each voxel is highly synchronized). However, by simply examining the change in the total voxel-wise connections between two brain regions across time, subtle effects may not be easily detectable. For example, one might imagine a scenario where there exists significant voxel-wise connectivity change over time between two large brain regions; however, due to both connectivity loss and gain in voxel pairings, the net change is close to zero. This would mislead an investigator to believe that no change has occurred. Therefore, to accurately characterize the change in the functional relationship over time between a pair of ROIs, connections between sub-regional-pairs that show significant change in their functional relationship strength must be detected, including both connection gain and loss. We define a sub-region as a cluster of spatially connected voxels within a ROI, where each voxel can have 6 neighbors in 3 dimensions. A sub-regional-pair consists of a sub-region from each of the two ROIs (see **Figure 3B**).

The goal of this study is to develop a novel approach for examining functional plasticity between two ROIs across time by detecting such significantly plastic sub-regional-pairs. In this article, the change in the strength of the functional relationship between two regions (either the entire ROI-pair or a constituent sub-regional-pair) across time will be referred to as plasticity. In particular, *positive plasticity* refers to situations where the strength of the functional relationship between two regions becomes stronger (i.e., two regions show an increase in synchronization over time); similarly, *negative plasticity* refers to cases where the strength of the functional relationship becomes weaker.

We introduce a novel voxel-wise evolutionary computation (EC) based procedure to document plasticity between an investigator defined ROI-pair across two fMRI-scan sessions that are separated by a finite duration of time; the procedure can be run for each subject separately, and it simultaneously utilizes the BOLD-fMRI data from both of the sessions. Of note, we choose a commonly used anatomical atlas for ROI selection (e.g., AAL atlas) and this space is refined over the course of the EC procedure based upon connectivity change. In this sense, the chosen ROI provides an initial constraint of the search space for the algorithm but the procedure is agnostic to the nature of the ROI. The procedure is data-driven, and detects sub-regional-pairs that exhibit significant positive and negative plasticity (see Section Objective Function). These sub-regional pairs are then used for developing a subject specific estimate of overall positive and negative plasticity. The challenge in detecting such plastic sub-regional-pairs is primarily due to the fact that many combinations of the connected subset (see Section Chromosome Encoding) of voxels from each ROI must be probed. Hence, for large ROI-pairs, examining different combinations of sub-regional-pairs becomes computationally very expensive. Therefore, we chose to use an evolutionary computation (EC) based approach. An advantage of using EC is that it is implicitly parallelizable (Rechenberg, 1971; Schwefel, 1974; De Jong, 2006; i.e., EC may be broken into multiple concurrent processes; this can drastically reduce total computation time) and has been widely used in handling problems of similar complexity in other research domains (Gruau and Whitley, 1993; Gruau, 1994; Kobayashi and Ohbayashi, 1999; Roy et al., 2013).

We illustrate (1) the insights gained by considering significantly plastic sub-regional-pairs and (2) the strength of this EC procedure in the study of brain plasticity. To do so, we examine the functional plasticity between four ROI-pairs in traumatic brain injury (TBI) and healthy control (HC) subjects with focus on large-scale networks that are now central to the cognitive neurosciences (e.g., DMN, ECN). Specifically, in a ROI-pair A-B, A is either DMN-frontal left or DMN-frontal right and B is either ECN-frontal left or ECN-frontal right, for a total of four ROI-pairs. The ROIs are defined using AAL atlas (Tzourio-Mazoyer et al., 2002); the brain regions constituting these ROIs are presented in Table S5. These ROIs have been chosen because there have been well documented functional changes in DMN and ECN post TBI (Bonnelle et al., 2011; Sharp et al., 2011; Venkatesan et al., 2014); these results may be used to validate the findings of the EC procedure. To examine the utility of this EC-based approach we conduct the following analyses:

1. *Analysis-1:* First, we use conventional ROI-averaging based procedure to define functional connectivity between the 4 ROI-pairs for each subject at two different time-points when BOLD-fMRI scans were conducted (see Section Subjects). Then we examine if the functional connectivity change can be detected across the two time-points at a group level for TBI and HC subjects. We hypothesize that this approach will detect little to no plasticity.
2. *Analysis-2*: Next, we define the strength of functional connectivity between a ROI-pair as the number of statistically significant voxel-wise functional connections that exist between the two regions. For a given voxel-pair, a significant connection indicates that the activities recorded at the two voxels are highly synchronized (have high correlation value). Thus, a great change in the number of statistically significant voxel-wise functional connections across time for a ROI-pair corresponds to a great change in the level of their synchronization (i.e., it represents significant plasticity). We then examine if this way of defining functional connectivity helps us to detect any plasticity effects in the TBI and the HC groups across two time-points when BOLD-fMRI scans were conducted (see Section Subjects). While we hypothesize that this approach will be able to detect some plasticity, we also hypothesize that a more sensitive procedure will detect greater plasticity.
3. *Analysis-3*: Next, we document the drawbacks of the simple voxel-based approach (defined in Analysis-2) to examine plasticity, and introduce the EC-based approach which is also built upon the basic idea of counting statistically valid voxel-wise connections between regions. However, the EC based approach makes use of an adaptive algorithm that probes for sub-regional-pairs that show significant decrease/increase in statistically valid voxel-wise connections across two time-points when BOLD-fMRI scans were conducted (see Section Subjects). Thus, the EC-based approach detects sub-regional-pairs within a ROI-pair that exhibit significant plasticity (the plasticity of each sub-regional-pair is either positive or negative). Using these sub-regional-pairs, an overall estimate of positive and negative plasticity will be derived for the given ROI-pair.

The EC approach involves some stochasticity, and therefore, it is very important to examine its performance across multiple independent runs. Since the primary goal of this article is to use the EC procedure to estimate the level of positive and negative plasticity between a ROI-pair, we document its performance by measuring the consistency of the plasticity estimates found across multiple independent runs. As a secondary measure of consistency, we also document the spatial overlap between the sub-regional-pairs found across independent runs.

## Materials and methods

### Data

#### Subjects

The study included 26 subjects, 14 individuals with moderate and severe TBI and 12 age and education matched healthy control participants (HCs; see Table 1 for demographic information). Individuals with TBI completed two separate MRI sessions ~3 and 6 months following the resolution of post-traumatic amnesia. HCs completed two separate MRI sessions separated by ~3–4 months to provide context for natural variation in healthy brain networks (see Table 1). For the purposes of this article, the two time-point fMRI-scan sessions will be referred to as session-1 and session-2. For the TBI sample, each testing session included both MRI data collection and cognitive testing. Moderate and severe TBI were defined using Glasgow Coma Scale (GCS) at the time of injury, whereby a score of 3–8 was indicative of severe injury and a GCS of 9–12 was indicative of moderate injury (Teasdale and Jennett, 1974). Two subjects who received a GCS of 14 and one subject who received a 24-h GCS of 15 were included because of either mental status change after the initial GCS scoring or the presence of significant brain injury documented through medical records. Subjects were excluded if they were receiving treatment for concomitant injuries (e.g., orthopedic injuries or injury to the spinal cord) that would make it difficult for them to remain still and comfortable in the MRI environment. For the purposes of this study where we aim to examine functional plasticity, we use a well-documented clinical sample with known network changes from 3–6 months post injury (Hillary et al., 2014; Rajtmajer et al., 2015).

**Table 1:** **Subject demographic information**.

	**Age**	**Education**	**Gender**	**GCS**	**Months post injury scan 1**	**Months post injury scan 2**
TBI mean (std)	26.07 (6.56)	13.42 (2.43)	7 M; 7 F	7.92 (4.96)	3.36 (0.74)	6.64 (1.45)
HC mean (std)	36.42 (15.25)	13.42 (1.88)	7 M; 5 F	N/A	N/A	N/A

Research was approved by the Institutional Review Board and the Office of Research Protections at the Pennsylvania State University. Individuals included in the study demonstrated some level of cognitive impairment. If an individual retained the ability to sign medical documents and/or function independently, then consent was accepted; if the individual was not functionally independent, then a caregiver's signature was required in addition to the subject's signature of assent.

#### Functional data

Study participants were scanned using one of three MRI machines, including a Philips Achieva 3T scanner in the Department of Radiology at Hershey Medical Center, Hershey, PA and two identical Siemens Magnetom Trio 3T scanners (Social, Life, and Engineering Sciences Imaging Center at the Pennsylvania State University in University Park, PA; Department of Radiology at Hershey Medical Center in Hershey, PA). Of the subjects, 7 were scanned on the Achieva 3T scanner, 5 subjects were scanned on the Magnetom Trio 3T scanner at the Pennsylvania State University, and 14 subjects were scanned on the Magnetom Trio 3T scanner at Hershey Medical Center. For repeat scanning, all subjects were scanned on the same machine across time points. Prior to scanning subjects were made aware of the importance of minimizing motion within the scanner which at times included pre-scanning preparation in the mock-scanning environment.

#### Data acquisition parameters

Anatomical images with a spatial resolution of 1.0 × 1.0 × 1.0 mm were acquired using an MPRAGE sequence: 2000 ms/2.03 ms/9° (repetition time (TR)/echo time (TE)/flip angle (FA), 256 × 256 mm^2^ field of view (FOV), and 256 × 256 acquisition matrix with 1 mm slices. Echo planar imaging (EPI) was used to examine the blood oxygen level dependent response for functioning imaging. Imaging parameters for EPI were 2000 ms/30 ms/90° (TR/TE/FA), 240 × 240 mm^2^ FOV, and 80 × 80 acquisition matrix with 4 mm slices.

#### Data preprocessing

Resting data were collected over the course of a 5 min period, resulting in 150 volumes of data. The first five volumes were removed from analyses to control for signal instability, resulting in a time series of 145 volumes. For all volumes bad slices were first repaired using the art-slice procedure, which is part of the ArtRepair toolbox (Mazaika et al., 2009). The volumes were then slice time corrected and realigned to the first volume using SPM8. Even after realignment, there remain important influences of motion. As recommended in the ArtRepair manual, this can be handled via Volume repair at the end of the preprocessing pipeline. Power et al. (2012) demonstrate that global signal regression is a powerful method for reducing the effects of motion. Given concerns published elsewhere (Murphy et al., 2009; Saad et al., 2012) we do not use this approach. Spike artifacts were eliminated using the despike filter available in the ArtRepair toolbox. Each subject's high-resolution (1 × 1 × 1 mm) TI image was co-registered to the mean functional image using SPM8. The co-registered T1 image was then segmented using SPM8, which produced a normalized (MNI space) gray matter image. The functional images were then normalized to the MNI space. The normalized functional volumes and the gray matter image were resliced, and the voxel dimensions were defined as 3 × 3 × 3 mm. The equivalence of voxel size allowed one to one mapping of voxels between a normalized functional and normalized T1 volumes, and provided a mechanism to extract gray matter signal (see the following section). To reduce the effect of ringing artifact (Lindquist and Wager, 2008) and to improve the signal to noise ratio, a 6 mm smoothing filter was applied. The CSF and the white matter nuisance signals were regressed out, and a bandpass filter of 0.01 to 0.12 Hz (Bassett et al., 2011; Rajtmajer et al., 2015) was applied using the CONN toolbox (Whitfield-Gabrieli and Nieto-Castanon, 2012). The removal of nuisance signal due to physiology and head motion is a developing issue receiving significant recent attention in the resting MRI literature (Power et al., 2012, 2014). Finally, the bad volumes (volumes with excessive motion related problems) were repaired using ArtRepair software (Mazaika et al., 2009).

#### Binary gray matter mask

We developed an average gray matter image using the normalized/resliced gray matter images of the control subjects at both time-points (12 gray matter images × 2 scan sessions = 24 gray matter images). Since the segmentation procedure assigns a probabilistic value to each voxel of the gray matter segmented file (i.e., the probability that the voxel belongs to the gray matter), we used only the voxels with probability value greater than 0.6 (Zalesky et al., 2012) to define a group averaged binary gray matter mask.

#### Defining ROIs

To determine the gray matter voxels that are part of DMN (left/right) and ECN (left/right), we first co-registered Automated Anatomical Labeling (AAL) atlas (Tzourio-Mazoyer et al., 2002) to the group averaged binary gray matter mask (See Section Binary Gray Matter Mask). This allowed us to label each gray matter voxel with a unique AAL identification number. Since the binary gray matter mask and the functional images are composed of voxels of the same dimensions and are normalized to MNI-space (See Section Data Preprocessing), the voxels have one to one correspondence between these two image modalities. Hence, the ROIs are defined using the gray matter voxels with AAL identification numbers that represent the brain regions that are part of DMN (left/right) and ECN (left/right). The brain regions constituting these ROIs are presented in Table S5.

### Developing subject-specific connectivity matrices

As discussed in the introduction, both analysis-2 (simple voxel-based approach) and analysis-3 (EC based approach) are based on counting statistically significant voxel-wise connections between the ROI-pairs. Thus given a ROI-pair A-B, we first create two voxel-level connectivity matrices for each subject: one for session-1 (*M*_1_) and one for session-2 (*M*_2_). For each session, the connectivity matrices are developed by performing a Pearson's correlation between the set of signals that are recorded from the voxels that are part of ROI-A to the set of signals that are recorded from the voxels that are part of ROI-B. For example, if ROI-A consists of 500 voxels and ROI-B consists of 1800 voxels, then the connectivity matrix will have a dimension of 500 × 1800. The correlation values that fail false discovery rate (FDR) test at a threshold of 0.05 are discarded; these cells in the connectivity matrices are set to 0.

For illustration purposes, for both analysis-2 and analysis-3 (see Introduction) we only consider positively correlated functional edges as we are interested in examining the change in positive synchronization between the four ROI-pairs. Therefore, we discard the cells that represent negative correlations (these cells are set to 0 as well). However, both the simple voxel-based approach and the EC process are not dependent on this choice, and an investigator may choose to use both positive and negative correlated edges. The remaining cells were set to 1. Since we binarized the voxel-wise connections, while computing the change in connectivity strength of a sub-regional-pair across two fMRI-scan sessions we only need to compute the total number of connections at session-1 and session-2 instead of storing two separate distributions of statistically significant (FDR corrected; See the paragraph above) voxel-wise correlation values for each session. This makes the process computationally very efficient (less memory required). Regardless of the memory requirement, if the session-1 and session-2 distributions of statistically significant voxel-wise correlation values are used for examining plasticity between a given sub-regional-pair across time, then additional statistical correction mechanisms may have to be implemented as the number of data-points in the distributions may not be the same. For example, in an extreme case, for one or both of the sessions the distribution may contain no data-points if there are no significant voxel-wise connections. Of course, it remains a possibility that the connection strengths may be used to examine plasticity, but for the sake of simplicity we chose to binarize the connectivity matrix. Even though we binarize and lose some information, the approach discussed in this article provides novel insights into connectivity changes which otherwise would be hard to detect with an averaging based approach.

### Evolutionary computation based approach to detect significantly plastic sub-regional pairs

Given an investigator defined ROI-pair, ROI-A and ROI-B (henceforth, we will refer to this ROI pair as A-B), the goal of the evolutionary search based procedure is to find significantly (see Section Objective Function) plastic sub-regional-pairs within A-B by simultaneously utilizing BOLD-fMRI data collected from two fMRI-scan sessions that are separated by a finite duration of time; in this article for both TBIs and HCs the sessions are separated by 3 months (See Section Subjects). Further, this procedure is designed such that it can be run for each subject separately.

The procedure is schematically shown in **Figure 2** and can be summarized as follows. For a given ROI-pair, A-B, we search for significantly plastic sub-regional-pairs in a recursive manner; at each recursion level an EC based search is conducted for a significantly plastic sub-regional-pair. At recursion level-1, if a significantly plastic sub-regional-pair is found then all the edges between the detected sub-regional-pair are stored in a file, and then discarded from the region (from both session-1 and session-2 data) so that the EC does not converge to this solution again and searches for another significantly plastic sub-regional-pair at the next recursion level. At recursion level-2, the evolutionary search procedure is applied to the remaining edges; the process repeats (recursion level-3, recursion level-4,….) until no significantly plastic sub-regional-pair can be found. That is, we terminate the entire procedure at the recursion level at which the EC based search fails to find a significantly plastic sub-regional-pair. As an example, if the procedure is run such that session-1 and session-2 data are intentionally made identical, then the EC based search fails to find a significantly plastic sub-regional-pair (as there are none) at recursion level-1, and hence the entire procedure terminates at recursion level-1. Using the set of significantly plastic sub-regional-pairs detected by the EC based procedure, we then develop estimates for positive and negative plasticity (see Section Estimating Positive and Negative Plasticity) for the ROI-pair. In the following paragraph, we discuss the EC-based search that is conducted at each recursion level.

Evolutionary algorithms are designed to mimic biological evolutionary processes to solve multi-modal and multi-dimensional optimization problems (Rechenberg, 1971; Schwefel, 1974; De Jong, 2006). In evolutionary computation (EC), a candidate solution (which is analogous to biological phenotype) for a given optimization task is encoded as an array of numbers (which is analogous to biological genotype/chromosome). If the array of numbers (the chromosome) directly represents the candidate solution, then it is referred to as a direct mapping, otherwise it is referred to as an indirect mapping (Gruau and Whitley, 1993; Gruau, 1994; Kobayashi and Ohbayashi, 1999; Roy et al., 2013).

For our purposes, given an investigator defined ROI-pair, ROI-A and ROI-B, a candidate solution represents one sub-region from each ROI. Henceforth, we will refer to this ROI pair as A-B. Each sub-region is composed of a connected set of voxels. Two voxels are said to be connected (or neighbors) if they share a common physical boundary, i.e., there is a path of length 1 between these two voxels. A set of voxels are said to be connected if there is a path between every pair of voxels in the set. The chromosome will not encode these voxels directly; instead, a compression scheme (see section Chromosome Encoding below) will be used to define a sub-region within each ROI. Thus we use indirect mapping in this work.

**Figure 1:**
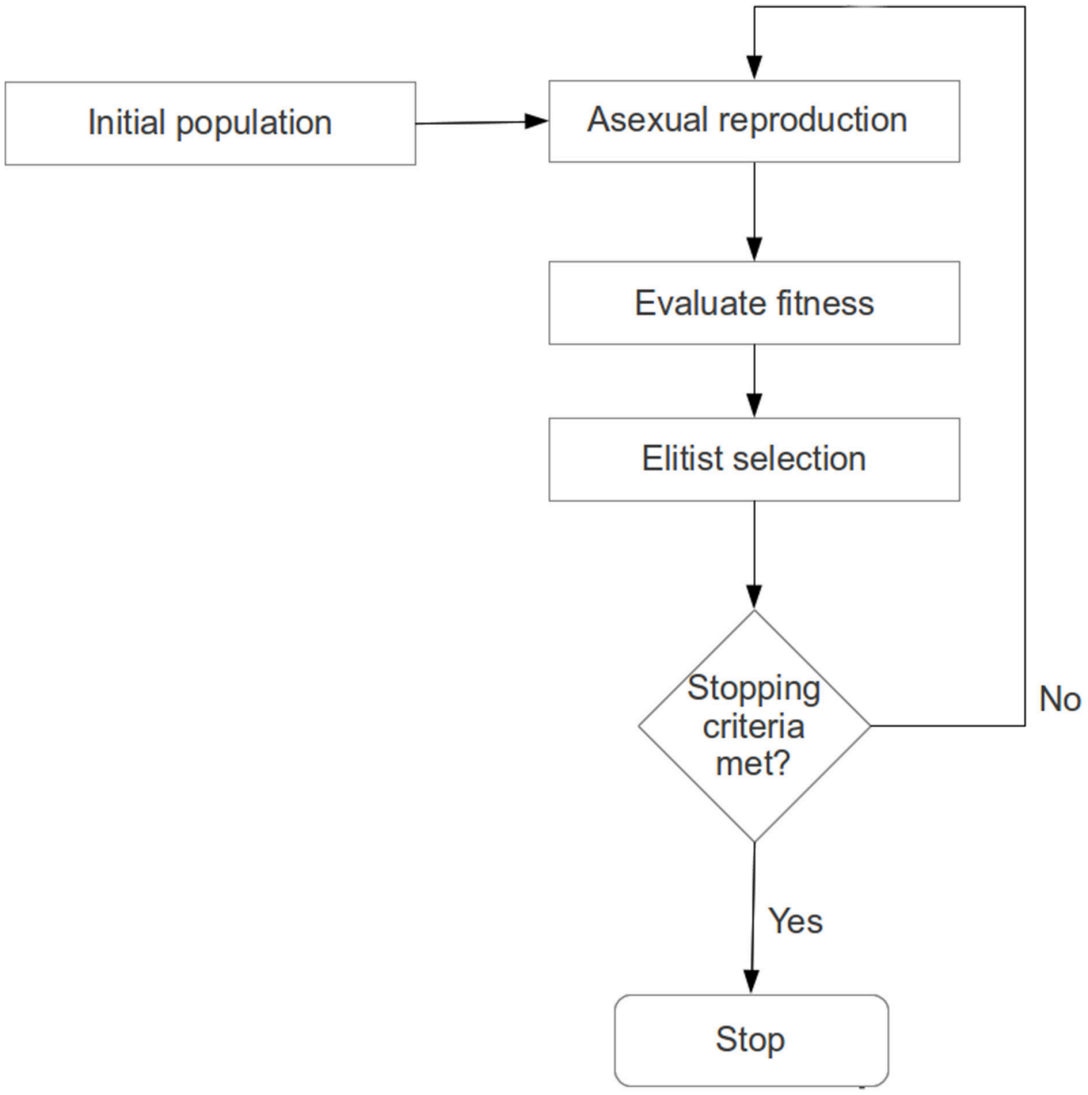
**The sequence of steps involved in the evolutionary computation based procedure**.

**Figure 2:**
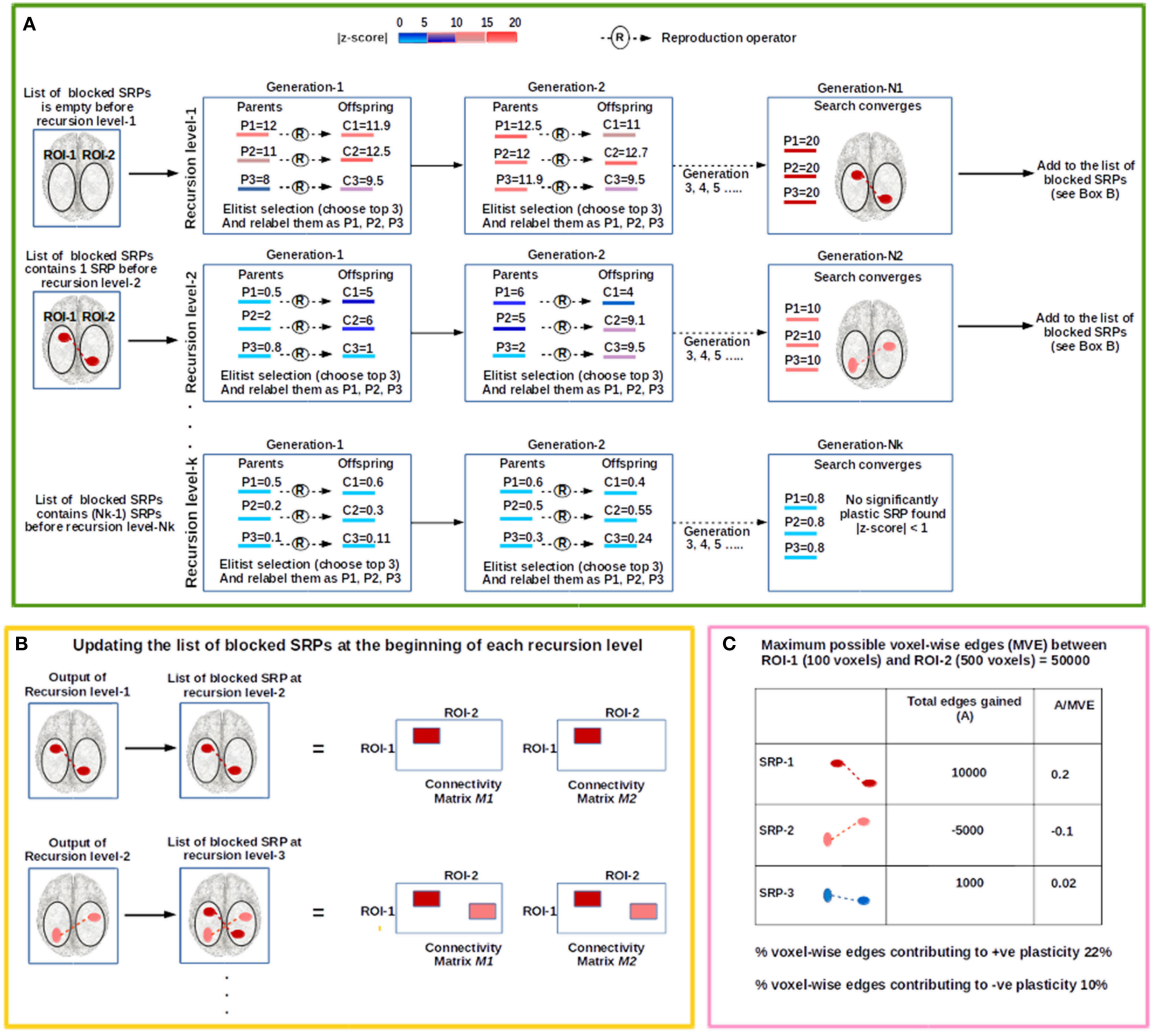
**The figure illustrates the steps involved in the evolutionary computation (EC) based procedure to examine plasticity between an investigator defined ROI-pair (shown as the oval shaped white regions on the brain image), ROI-1 and ROI-2. (A)** The procedure begins at recursion level-1 where the evolutionary search for a significantly plastic sub-regional-pair (SRP) is conducted. In this illustration, the population size (number of parental chromosomes) is 3, and each parental chromosome (P1, P2, and P3) produces 3 offspring (C1, C2, and C3). Based on population-elitist selection the top 3 chromosomes are chosen for the next generation, and relabeled as P1, P2, and P3 in a descending order of fitness. The color of each chromosome represents its fitness (|Z-score|); the higher the fitness of the chromosome, greater is the change in the connectivity strength between the sub-ROIs that the chromosome encodes. At the end of recursion level-1, a significantly plastic SRP is detected (SRP-1; shown in dark-red) and the chromosome encoding this SRP is stored in a file. SRP-1 is then added to the list of blocked SRPs, and therefore, at the beginning of recursion level-2 the list contains one blocked SRP; EC will not converge to this solution at recursion level-2. At recursion level-2 a new search begins, and SRP-2 (show in light-red) is detected. For recursion level-3, SRP-1 and SRP-2 are blocked. The recursive process stops at recursion level-*K* when no significantly plastic SRP is found. **(B)** To block a SRP all the voxel-wise edges between the SRP are deleted from connectivity matrices (See Section Developing Subject-Specific Connectivity Matrices), *M1 and M2 (*shown as colored rectangular patches). **(C)** We illustrate an example consisting of 3 significantly plastic SRPs to show how plasticity estimates are derived.

At a given recursion level, an evolutionary search for a significantly plastic sub-regional-pair will begin with a randomly generated population of chromosomes, each representing a sub-regional-pair within A-B. Each chromosome will then produce an offspring chromosome using an asexual (single parent) reproduction operator (Fogel, 1999, 2003; Davoian et al., 2006) (See Section Reproduction Operator for details). The reproduction operator ensures that an offspring shall encode a sub-regional-pair that has some spatial similarity (many common voxels) to the sub-regional-pair encoded by the parent (see section Chromosome Encoding); this process ensures that the search is not random. The fitness value (or quality of each solution) of a chromosome (parent and offspring) will be evaluated based on the level of plasticity (see objective function below) that the sub-regional-pair exhibits across 2 fMRI-scan sessions. That is, if the number of voxel-wise functional connections between the sub-regional-pair changes significantly across two sessions (session-1 and session-2), then the sub-regional-pair is highly plastic (see **Figure 4**). To ensure that the best solutions are preserved, a population-elitist selection (Eshelman, 1991; Eshelman and Schaffer, 1991) procedure will be implemented in each generation to choose the fittest chromosomes (parents and offspring combined) for the next generation; the population size in every generation will remain constant (see Section Evolutionary Search Parameters and **Figure 2**). That is, in population-elitist selection all the offspring and parents are pooled together and top 50% best solutions are chosen. As a result of this, even the fitness landscape near poor solutions gets the chance to be explored across generations. Hence, this form of selection maintains good diversity in the population, and therefore, provides a mechanism to prevent the evolutionary search from premature convergence (Eshelman and Schaffer, 1991). The reproduction-process, fitness-evaluation, and population-elitist selection steps will be repeated until the stopping criteria (see Section Evolutionary Search Parameters) are met (see Figures 1, 2).

The basic idea behind this evolutionary search can be explained as follows. Since the fittest candidate solutions (i.e., fittest chromosomes) survive to reproduce, the space around the sub-regional-pairs encoded by those fittest candidates are searched thoroughly over generations as a result of the reproduction operation, thereby increasing the likelihood of discovering significantly plastic sub-regional-pairs. This entire procedure is referred to as “evolutionary” because it holds similarity to natural evolution where the phenotypes that are suited for survival propagate along the generations and eventually become prevalent in the population; likewise our evolutionary search eventually converges to sub-regional-pairs (the artificial phenotype) that represent strong plasticity. Below, we discuss the details of this procedure.

#### Chromosome encoding

**Figure 3:**
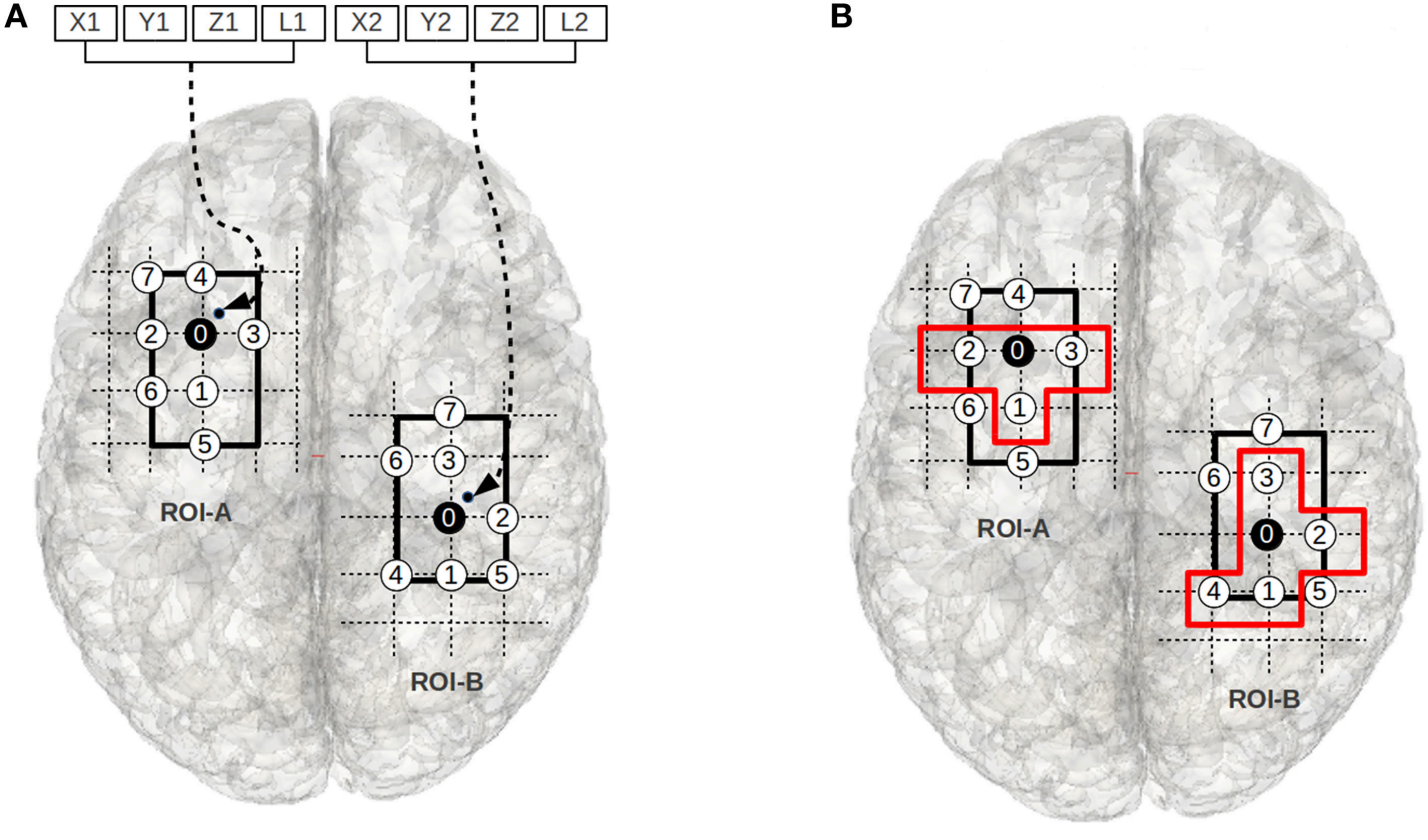
**(A)** The figure illustrates the chromosome structure used for the evolutionary-search. The voxels 0–7 in the left grid and the voxels 0–7 in the right grid represent a ROI pair. The chromosomal-variables X1, Y1, and Z1 encode a point (small black-dot) within the smallest cuboid (the thick rectangular boundary in left grid) encapsulating ROI-A, and X2, Y2, and Z2 encode a point (small black-dot) within the smallest cuboid (the thick rectangular boundary in right grid) encapsulating ROI-B. The closest gray matter voxels to these points are chosen as the root voxels (i.e., voxel 0 in both ROIs). The circles with numbers represent gray matter voxels. The circles are sequentially numbered using BFS strategy w.r.t the root voxel; the voxels with lowest z-axis values are numbered first, followed by the voxels with lowest y-axis values, and then lowest x-axis values. L1 and L2 define the number of voxels chosen around the root node from ROI-A and ROI-B using a BFS strategy to form the sub-regional-pair; the sub-regional-pair also includes the root node. **(B)** We illustrate an example of a sub-regional-pair where L1 = 3 and L2 = 4. The voxels within the orange boundaries represent the sub-regional-pair.

A chromosome indirectly encodes a sub-regional-pair within A-B (see Figure 3B). We will explain the chromosome encoding using a specific example shown in Figure 3A. In Figure 3A, the voxels 0–7 in region-A (the left grid) and voxels 0–7 in region-B (the right grid) represent a ROI pair A-B; for the purpose of clarity we have not shown the edges that exist between these regions. The thick black boundaries represent the smallest cuboids, cuboid-A and cuboid-B, that respectively encapsulate the regions A and B. For the ease of explanation, in Figure 3A we have considered a special case where the ROI pair A-B has no width in the Z-direction (the direction pointing out of the page), and therefore, the cuboid encapsulating them appear as a rectangle.

The objective of the evolutionary search is to find a pair of sub-regions (i.e., a sub-region within cuboid-A and a sub-region within cuboid-B) that qualifies as significantly (see Section Objective Function) plastic where each sub-region is composed of a set of connected voxels. Two voxels are said to be spatially connected (neighbors) if they share a common boundary: a cubic-voxel can have 6 possible neighbors. The first 3 locations of the chromosome *(X1, Y1*, and *Z1)* encode the coordinate of a point (shown as a small black-dot in Figure 3A) inside cuboid-A. The voxel that is closest to this point and part of the ROI is chosen as the root node (the black node in Region-A, i.e., voxel 0), and the fourth component of the chromosome (an integer *L1*) determines the number of neighbors that are selected for membership in the sub-region. The neighbors are chosen using a breadth first search (BFS) strategy. The variables *X2, Y2, Z2*, and *L2* (see Figure 3A) encode a sub-region within cuboid-B in an analogous way. We note that while we use this BFS approach in this work, alternative approaches for choosing the neighboring voxels exist. The motivation for choosing the BFS strategy in this work is because it imposes the constraint that the sub-regional-pair must be composed of a connected set of voxels.

In some cases the BFS search may identify multiple candidate voxels for inclusion. In these cases we arbitrarily prioritize voxels with the smallest z-axis value, then similarly with the y-axis and then x-axis values. For example, in Figure 3B, if *L1* = 3, then the voxels 1, 2, 3, and the root node (voxel 0) represent the sub-region within ROI-A. While this choice influences the shapes of candidate sub-regional-pairs, the shape of the solutions carries little meaning in the absence of structural information. Rather, the location and general size of the significantly plastic sub-regional-pairs is of particular interest specifically because this information may provide structural insight.

#### Reproduction operator

In the context of the current work, we require a reproduction operator that allows the offspring chromosomes to encode sub-regional-pairs that have some spatial similarity to the sub-regional-pairs encoded by the parental-chromosomes. As many operators can be developed to accommodate this requirement, we decided to develop a simple mutation based reproduction operator (i.e., a single parent reproduction operator, See Figure 2) that allows an investigator to easily control the level of similarity between a parent and an offspring chromosome; this operator is similar to mutation based reproduction operators used in evolutionary programming (Fogel, 1999, 2003; Davoian et al., 2006). If offspring are made to be too similar to the parents, then the search is less exploratory. On the other hand, if the offspring are too different from the parents then the search becomes quite random. An appropriate level of exploration is often problem-specific, and it is generally determined by adjusting the exploration level till consistent good solutions are discovered across multiple independent runs. We have explained the reproduction operator below.

A parental-chromosome (of size 8 as discussed above) is given as follows:

Parent = {X1, Y1, Z1, L1, X2, Y2, Z2, L2}

An offspring will be generated by producing a random number for each of the eight positions within a bound defined by the offset-parameters *{dx1, dy1, dx1, dl1, dx2, dy2, dz2, dl2}*. For example, the value of X1 for a child, *X1*_*c*_, will be determined by the value of X1 for its parent*, X1*_*p*_, such that it falls in the range [*X1*_*p*_−*dx1, X1*_*p*_+*dx1*].

Here, we note that even though the evolutionary search begins with a population of randomly generated chromosomes encoding various combinations of sub-regional-pairs, the reproduction operation along with the population-elitist-selection procedure causes the search trajectory to tend toward sub-regional-pairs that are highly plastic.

#### Objective function

Given a candidate solution (a sub-regional-pair encoded by a chromosome), the change in the functional relationship (plasticity) that occurs between 2 time points (i.e., 2 scan sessions) is defined using the number of connections that exist between the sub-regional-pair at session-1 (*NC*_1_), the number of connections that exist between them at session-2 (*NC*_2_), and the total possible connections (*TC*) that can exist between them. The values *NC*_1_ and *NC*_2_ can be easily derived by counting the total number of cells that have the value 1 in the connectivity matrices, *M*_1_ and *M*_2_ (see Section Developing Subject-Specific Connectivity Matrices), respectively.

Since the connections are binary (they exist or they do not), the objective function is defined as the Z-score of a binomial test performed using the fraction of connections between the candidate sub-regional-pairs existing at session-1 and session-2. The objective function is defined as follows.

(1) $$\begin{array}{r} P_{\text{1}}=NC_{\text{1}}∕TC \end{array}$$

Where P_1_is the fraction of connections that exist between the sub-regional-pair at session-1.

(2) $$\begin{array}{r} EC_{\text{2}}=P_{\text{1}}^{*}TC \end{array}$$

Hence,

(3) $$\begin{array}{r} EC_{\text{2}}=NC_{\text{1}} \end{array}$$

where *EC*_2_ is the expected number of connections at session-2 between the sub-regional-pair established using session-1 data.

The standard deviation for the binomial distribution established using session-1 data will be defined as:

(4) $$\begin{array}{r} SD=Sqrt\left[TC^{*}P_{\text{1}}^{*}\left(1-P_{\text{1}}\right)\right] \end{array}$$

The fitness of the candidate solution will then be defined as the absolute (signs neglected) Z-score of the session-2 connection based on the binomial distribution established using session-1 data. That is,

(5) $$\begin{array}{r} Fitness=Z-score=|\left(NC_{\text{2}}-EC_{\text{2}}\right)∕SD| \end{array}$$

Thus a candidate solution with higher absolute Z-score will be more fit than a candidate solution with a lower absolute Z-score. That is, a highly fit candidate solution would represent sub-regional-pair with high level of plasticity (i.e., such sub-regional-pairs are significantly plastic).

**Figure 4:**
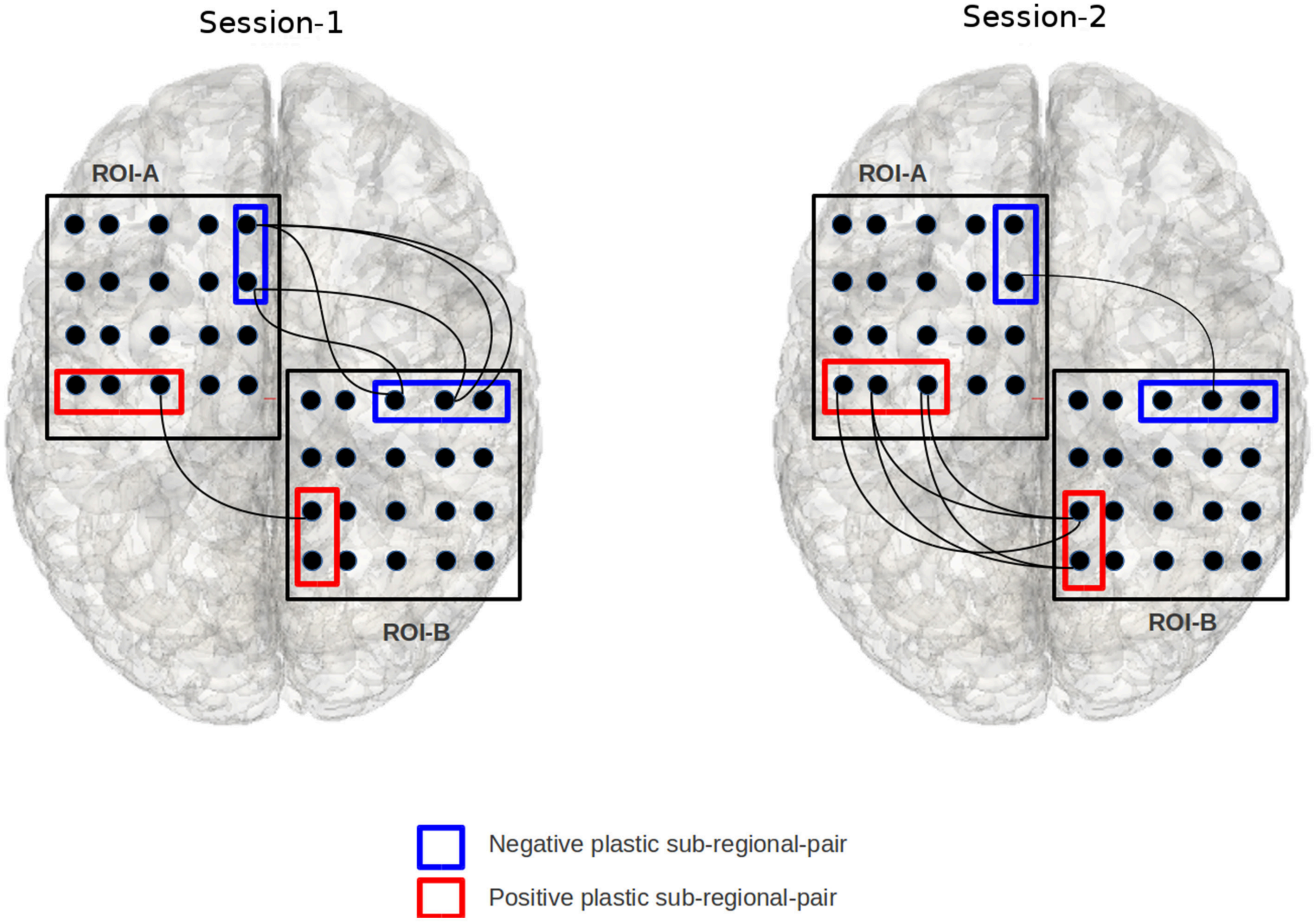
**The figure illustrates an example of a positively and negatively plastic sub-regional-pair**. In this figure, session-1 and session-2 refers to two fMRI-scan sessions that are separated by finite duration of time; chronologically, session-2 occurs after session-1. Edges indicate a voxel from ROI-A is significantly correlated with a voxel from ROI-B. The red sub-regional-pair gains functional edges from session-1 to session-2 (hence positive plastic), while the blue sub-regional-pair shows decline in the number of functional edges (hence negative plastic). The binomial test based objective functional detects such plastic sub-regional-pairs.

Note that the fitness function disregards the sign of the Z-score, as the purpose of the evolutionary search is to simply find significantly plastic sub-regional-pairs while disregarding the direction of plasticity. However, once the evolutionary process finishes running and locates the significantly plastic sub-regional-pairs, we run a simple *post hoc* analysis to determine the direction of the change according to the sign of the Z-score. This way we are able to detect both positively and negatively plastic sub-regional-pairs (Figure 4).

#### Evolutionary search parameters

**A. Criterion for stopping the search for any new significantly plastic sub-regional-pair** For a given investigator defined ROI-pair, A-B, the evolutionary search is run recursively until no new significantly plastic sub-regional pairs can be found. That is at a given recursion level, if an evolutionary search produces a solution with the objective function value less than 1 (i.e., Z-score less than 1), then the evolutionary search terminates, and no new search for a significantly plastic sub-regional-pair is initiated. Typically the EC based search starts by detecting plastic sub-regional-pairs with very high Z-scores (>>1). Therefore, for all practical purposes, when the evolutionary search starts producing solutions with a Z-score close to 1 (or less), most highly plastic sub-regional-pairs have already been detected. Of course, there is a possibility that the evolutionary search at a given recursion level may have converged to a poor solution. To confirm that no more good solutions (significantly plastic sub-regional-pairs) exist, either multiple runs should be executed or the search should be run with a sufficiently large population size. For the current work we chose the latter approach. The population size and other evolutionary parameters are discussed below.

**B. Criteria for stopping the evolutionary search** At a given recursion level, the evolutionary search is terminated when one of the following occurs:

1. Complete convergence (all chromosome encode the same solution).
2. The objective function value does not improve over a span of 100 generations. For a more rigorous search, one may choose this value to be much higher. In context of the present study, this value provided a good balance between runtime and ability of the procedure to detect statistically significant plastic sub-regional-pairs.

**C. Evolutionary search parameters** Below, we summarize the evolutionary search parameters:

1. Population size is set to 400.
2. Reproduction parameters, *dx1, dy1, dx1, dx2, dy2*, and *dz2*, were set to 6 mm; the parameters *dl1* and *dl2*, were set to 4. This forced the mutation operator to choose a root-voxel for an offspring not more than two voxels away from the parent root-voxel (the voxel size of preprocessed functional image is 3 × 3 × 3 mm ^3). As above, these values were chosen to provide a balance between consistent good solutions and run time; automatic tuning of these parameters could in principle be used to further optimize this approach.
3. Given a ROI-pair A-B, in our experiments we allowed the chromosomal-parameters *x1, y1, z1* to point to any spatial location within the smallest cube encapsulating ROI A, and *x2, y2, z2* to point to any spatial location within the smallest cube encapsulating ROI B. The chromosomal-parameters, *L1* and *L2* were allowed to take any value at a step size of 5 between 64 (4 × 4 × 4 voxels) to the total number of voxels in ROI A and ROI B, respectively. Both the DMN regions consisted of ~500 voxels, and each ECN region consisted of about 1800 voxels.

#### *Post hoc* correction for multiple tests

In reference to the objective function discussed above, a set of significantly plastic sub-regional-pairs detected using EC represents an array of statistically significant Binomial tests. Therefore, we perform Bonferroni correction to establish corrected *p*-values for the sub-regional-pairs; only the sub-regional-pairs with corrected *p*-values less than 0.05 are considered valid. In this article we chose Bonferroni correction because we wanted to be more conservative in our estimate of the plasticity levels of individuals with TBI and controls to examine robustly if there was heightened or diminished connectivity in TBI cases as compared to controls. Depending on context, other (e.g., data-driven) approaches may be appropriate; see for instance Derrac et al. (2011).

#### Estimating positive and negative plasticity

Using the set of significantly plastic sub-regional-pairs that qualify *post-hoc* correction (see Section *Post hoc* Correction for Multiple Tests), we evaluate the percentage of total voxel-wise connections that contribute to positive and negative plasticity. For example, as shown in Figure 2 (Box C), let us consider an investigator defined ROI-pair, ROI-1, and ROI-2, where ROI-1 is composed of 100 voxels and ROI-2 is composed of 500 voxels. Therefore, at most there can be 50000 voxel-wise connections between ROI-1 and ROI-2. Let us also consider that using the EC based procedure (including *post-hoc* correction) we detected 3 significantly plastic sub-regional-pairs: SRP-1, SRP-2, and SRP-3. SRP-1 and SRP-3 were positively plastic and gained 10000 and 1000 voxel-wise connections from session-1 to session-2, respectively, while SRP-2 lost 5000 connections from session-1 to session-2. Thus, the percentage voxel-wise connections contributing to positive plasticity is [(10000+1000)^*^100]/50000% = 22% and percentage voxel-wise connections contributing to negative plasticity is [5000^*^100]/50000% = 10%.

## Results

### Averaging based approach

**Figure 5:**
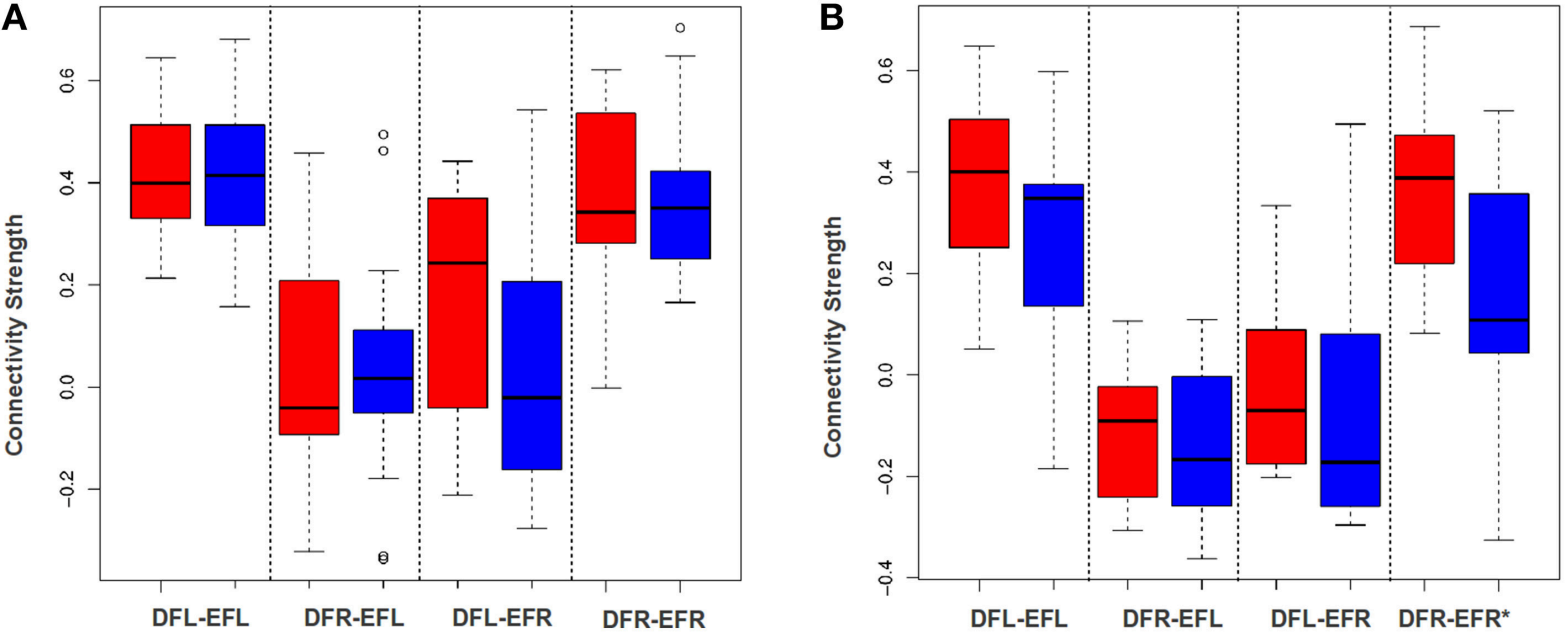
**DFL, DFR, EFL, and EFR, stand for default mode frontal-left, default mode frontal-right, executive frontal-left, and executive frontal-right, respectively. (A)** The box plot illustrates the distribution of the connectivity strength (correlation between ROI-wise averaged signal) of the TBI-subjects at session-1 (red) and session-2 (blue) for all the four ROI-pairs. **(B)** The box plot illustrates the distribution of the connectivity strength (correlation between ROI-wise averaged signal) of the control cases at session-1 (red) and session-2 (blue) for all the four ROI-pairs. Using paired *t*-test (after Fisher z-transforming the correlation values), the difference between the group level means at 2 sessions was found to be insignificant for the TBIs. For the controls, except for the fourth ROI-pair, plasticity for rest all ROI-pairs was insignificant.

To examine the effectiveness of the averaging based approach to detect plasticity, for each subject for each session we developed an average signal for all the four ROIs: DMN-frontal left/right and ECN-frontal left/right. Then we established the functional connectivity strength between all the four ROI-pairs for each subject for each session using Pearson's correlation coefficient. We performed a paired *t*-test of Fisher z-transformed correlation values to examine if this approach can detect group level plasticity (change in functional connectivity strength between session-1 and session-2) for the TBIs and the HCs. As shown in Figures 5A,B this approach failed to detect plasticity. Only the functional relationship between DMN-frontal right and ECN-frontal right in HCs was found to be significant. This supports our hypothesis that the averaging based approach fails to detect plasticity.

### Simple voxel-level approach

**Figure 6:**
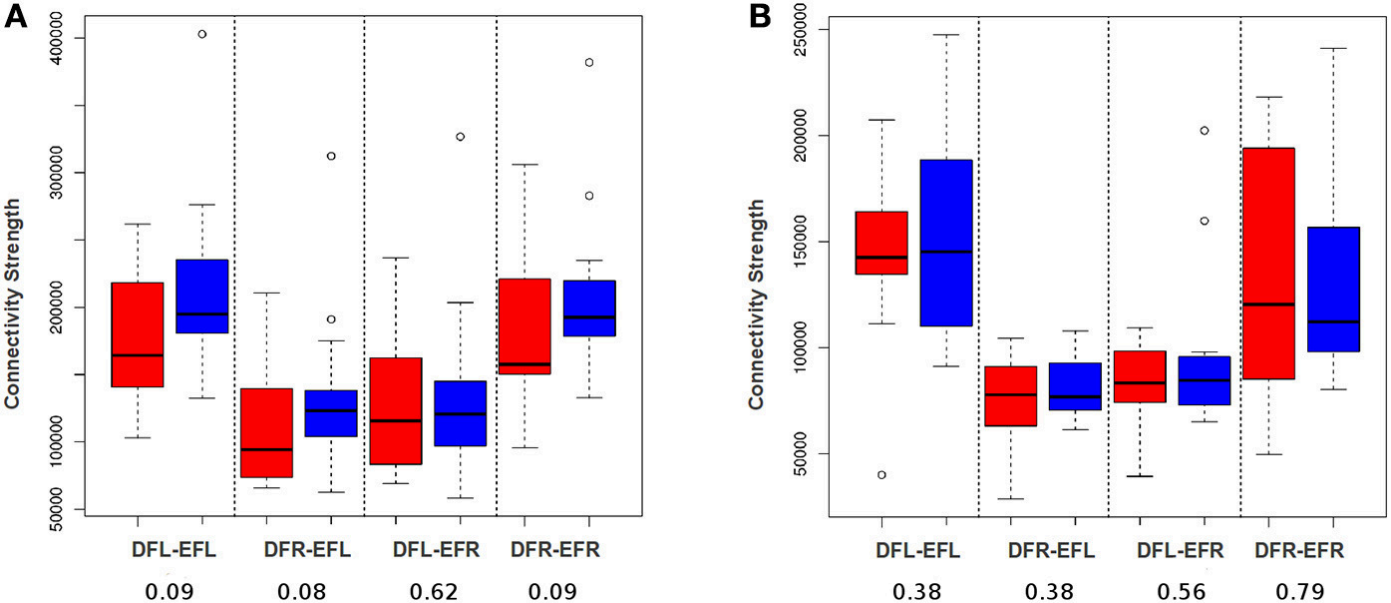
**DFL, DFR, EFL, and EFR, stand for default mode frontal-left, default mode frontal-right, executive frontal-left, and executive frontal-right, respectively**. Connection strength is defined as the number of voxel-wise functional edges between the ROI-pairs. **(A)** The box plot illustrates the distribution of connection strength of TBI-subjects at session-1 (red) and session-2 (blue) for all the four ROI-pairs. **(B)** The box plot illustrates the distribution of connection strength of control cases at session-1 (red) and session-2 (blue) for all the four ROI-pairs. Using paired Wilcoxon signed-rank test, the difference between the group level means at 2 sessions was found to be insignificant for TBIs and controls for all the 4 ROI-pairs. However, in contrast to averaging analysis (see Figure 5) there seem to be some signs of plasticity for TBI at a *p*-value of 0.1.

In order to demonstrate that a voxel-based approach will be able to detect plasticity better than an averaging based approach, we developed a simple edge count based procedure. That is, for each subject for each session, a connectivity matrix was developed for each ROI-pair (see Section Developing Subject-Specific Connectivity Matrices developing subject specific connectivity matrix). The total number of functional edges between a pair of ROIs for each subject at each session was evaluated by considering the connectivity matrices entry by entry and counting the cells that have the value 1. Using the total edge count we performed paired Wilcoxon signed-rank test for all the four ROI-pairs for HC and TBI group (see Figure 6); we chose a non-parametric test (Wilcoxon test) because based on Shapiro–Wilk tests we found the data was not consistently normal across all 4 ROI-pairs and across 2 time-points. As shown in Figure 6B, no plasticity was detected for the controls. For the TBI cases, the tests were insignificant at *p* = 0.05. However, at *p* = 0.1, three of the four ROI-pairs showed some signs of plasticity (see Figure 6A). This supports our hypothesis that a voxel-level procedure will be better able to capture plasticity than averaging based approaches. However, we anticipate that this simple voxel-level procedure may be underestimating the level of plasticity as it does not differentiate between positive and negative plastic sub-regional-pairs.

### Evolutionary computation based approach

#### Examining functional plasticity

**Figure 7:**
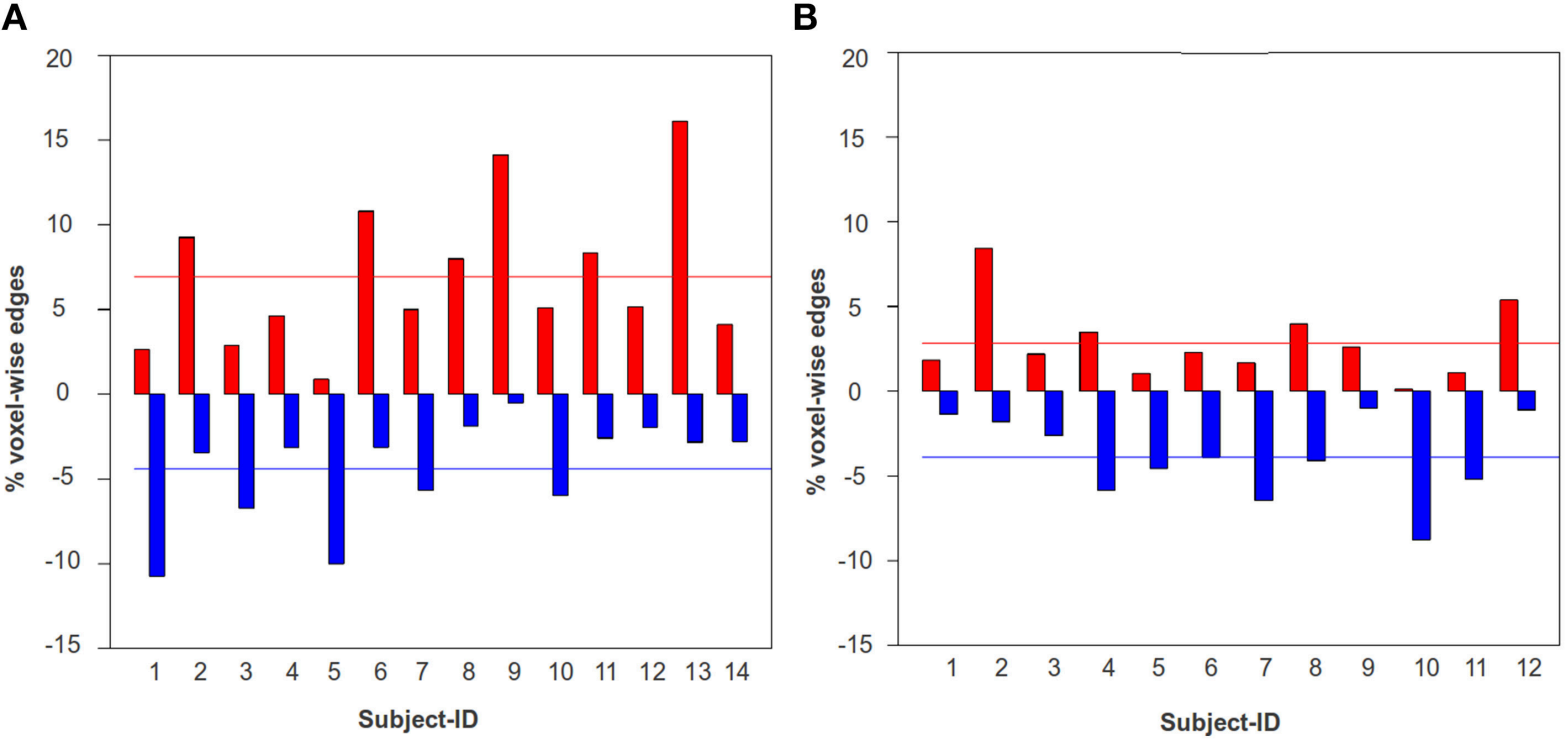
**The percentage of total voxel-wise connections between DMN-frontal right and ECN-frontal right that contributed to statistically significant positive (red) and negative (blue) plasticity**. A similar trend was observed for the ROI-pairs, DMN-frontal left and ECN frontal left, and DMN-frontal right and ECN frontal left. **(A)** The bar-graph illustrates the percentage of total voxel-wise edges that contributed to positive (red) and negative (blue) plasticity for each TBI subject. **(B)** The bar-graph illustrates the percentage of total voxel-wise edges that contributed to positive (red) and negative (blue) plasticity for each HC subject. The red and the blue horizontal lines indicate the mean positive and negative plasticity level across a group, respectively. The mean positive plasticity for the TBI was significantly higher than the controls.

In Figure 7, we have shown the percentage of total voxel-wise connections that contributed to positive and negative plasticity for each TBI-subject and the HCs between the ROI pair, DMN-frontal right and ECN frontal right. As illustrated in the figure the mean positive plasticity level of the TBI was found to be significantly higher than the HCs (*p* = 0.009). A similar trend was observed for the ROI-pairs, DMN-frontal left and ECN frontal left (*p* = 0.046), and DMN-frontal right and ECN frontal left (*p* = 0.06); the group-level comparison of the mean positive plasticity for each ROI-pair was conducted using Wilcoxon rank sum test. We chose a non-parametric test (Wilcoxon test) because based on Shapiro–Wilk tests we found the data was not consistently normal across all 4 ROI-pairs.

This finding is consistent with previous findings that suggested such heightened functional connectivity in large scale networks in TBI subjects (Nakamura et al., 2009; Bonnelle et al., 2011; Hillary et al., 2011a; Caeyenberghs et al., 2012). Figure 7 thus suggests that while there is significant amount of positive and negative functional plasticity in the HC brain, the TBIs show more positive plasticity across time. Knowing the positive and negative plasticity level for HCs can be very useful for establishing thresholds for defining a healthy brain, and hence can be used for developing diagnostic systems for detecting neurological illnesses/insults.

To examine the consistency of the EC procedure in estimating plasticity across multiple independent runs, we ran 10 experiments per subject per ROI-pair. We then evaluated the standard deviation of the plasticity estimates (% voxel-wise connections contributing to positive and negative plasticity) for each subject. The standard deviation of the plasticity estimate obtained across multiple runs was very low (typically < 0.5%), thus highlighting the consistency of this approach (see Tables S1–S4).

The EC procedure was run on a serial mode (processor information: Intel(R) Xeon(R) CPU E5-2670 0 @ 2.60 GHz, 2 GB memory), and we developed an estimate of the runtime for each ROI-pair by averaging across 260 experiments (10 experiment × 26 subjects); the runtime results are presented in Table S6. The average runtime was ~15 min for four ROI-pairs. Because EC is implicitly parallelizable, the runtime can be significantly reduced using parallel programming. The EC procedure was performed in the MNI space. While the EC procedure can be run in the native space, for our purposes, normalization was required for 2 reasons. First for examining functional relationship between regions, it required extraction of all gray matter voxels in the brain and most segmentation algorithms perform some degree of normalization (e.g., SPM8) and hence implicitly require re-sampling. Second, in order to examine connectivity changes at separate time points simultaneously, identification of equivalent regions was necessary and this was achieved via normalization. Since the voxel size of the functional image before (3 × 3 × 4 mm) and after (3 × 3 × 3 mm) normalization was very similar we anticipate that the computational runtime presented in Table S6 should remain roughly the same.

**Figure 8:**
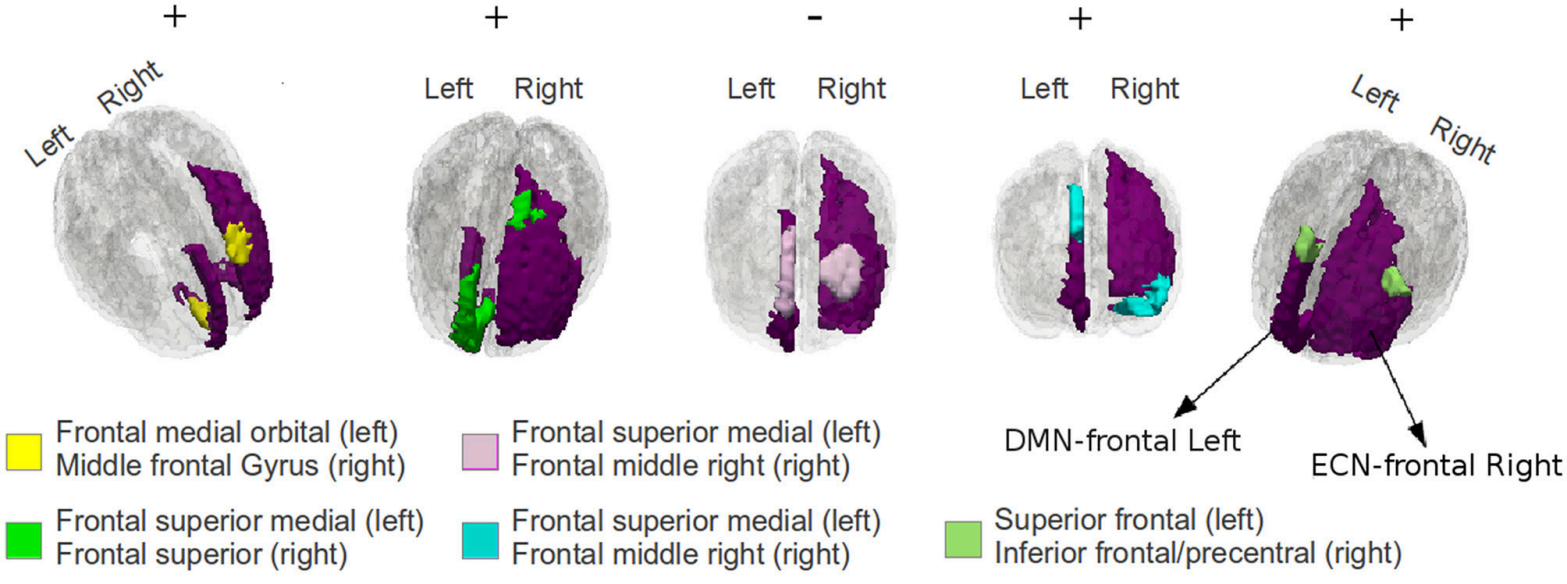
**An illustration of the first 5 significantly plastic sub-regional-pairs that were detected by EC based procedure between DMN-frontal left to ECN-frontal right for TBI subject-11; the DMN and the ECN regions have been colored purple**. The plastic sub-regional-pairs are colored yellow, green, pink, blue, and light blue (from left to right). The sub-regional-pairs are much smaller than the ROI-pair, and therefore, it may be hard to detect such subtle plasticity effects using a conventional averaging based approach. The +∕−ve signs indicate the plasticity direction.

In Figure 8, we have shown some significantly plastic sub-regional-pairs that were detected between DMN-frontal right to ECN-frontal left for TBI subject-11; for illustration purposes we have only shown the first 5 highly plastic (out of 41) sub-regional-pairs detected by the EC approach. The figure illustrates that sub-regional-pairs contributing to plasticity may be much smaller than the ROI-pair. The figure also illustrates that within a ROI-pair there may be both positively plastic sub-regional-pairs and negatively plastic sub-regional-pairs.

#### Overlap analysis

In order to test if the evolutionary process detects the same set of significantly plastic sub-regional pairs across multiple independent runs, per subject per ROI-pair the overlap analysis was conducted for 10 pair of independent runs.

The overlap between two sub-regional-pairs (a sub-regional-pair from run-1 and a sub-regional-pair from run-2) was defined using the number of common voxels they share; the overlap score was defined using the Sorenson-dice index (Sorenson, 1948). The Sorenson-dice index ranges from 0 to 1, with 0 indicating no overlap and 1 indicating complete overlap. For a given pair of independent runs, run-1 and run-2, the maximum spatial overlap score for each significantly plastic sub-regional-pair detected in run-1 was evaluated by comparing them with the significantly plastic sub-regional-pairs detected in run-2. In this way each sub-regional-pair from run-1 received an overlap score. Similarly, the maximum spatial overlap score for each sub-regional-pair detected in run-2 was evaluated by comparing them with run-1 sub-regional-pairs. The mean of the maximal overlap scores of all sub-regional-pairs (run-1 and run-2 included) was used for defining the overall overlap between the 2 runs. This procedure was repeated for all 10 pair of runs, per subject per ROI-pair and then the mean and standard deviation of the overlap score was established. As shown in Table 2A the overlap score was about 0.8. Also, we found no significant difference (*t*-test and Wilcoxon rank sum test, *p* > 0.05) between the Sorenson scores of the TBI and HC.

**Table 2A:** **Sub-cluster-pair overlap analysis based on Sorenson score**.

**ROI-Pair**	**TBI mean(stdev)**	**Control mean(stdev)**
DMN frontal-left to ECN frontal-left	0.79 (0.02)	0.80 (0.03)
DMN frontal-right to ECN frontal-left	0.80 (0.02)	0.80 (0.02)
DMN frontal-left to ECN frontal-right	0.80 (0.02)	0.79 (0.02)
DMN frontal-right to ECN frontal-right	0.82 (0.02)	0.82 (0.01)

We also performed overlap analysis using the adjusted rand index (ARI; Vinh et al., 2009). In this context, the ARI considers the set of voxel-pairs between the ROI-pair and measures the overlap between 2 different sets of labelings (produced by 2 independent EC runs), which we denote *T* and *U*. The set *T* has (*N*_*T*_ + *1*) labels representing *N*_*T*_ sub-regional-pairs and a label for voxel-pairs that are not part of any significantly plastic sub-regional pair. Similarly, the set *U* contains (*N*_*U*_ + *1*) labels. Here, each voxel-pair that exists in one of the *N*_*T*_ significantly plastic sub-regional-pair is assigned a corresponding value from {1,2,…,*N*_*T*_}; voxel-pairs that are not part of a significantly plastic sub-regional-pair are assigned a value of 0. This process is repeated for the *N*_*U*_ significantly plastic sub-regional-pairs identified in the second set of labelings. The ARI compares these labelings in such a way as to be invariant to label permutations. If the ARI is 1 then the overlap is perfect (i.e., the labelings are identical), and if the ARI is 0 then any observed overlap is due to chance. Thus the ARI metric examines how consistently the voxel-pairs are grouped into sub-regional-pairs across independent runs.

Per subject per ROI-pair, the ARI metric was calculated for 10 pair of independent runs. In Table 2B, we have summarized the mean and the standard deviation of the ARI score for the TBI and the HC group for all four ROI-pairs. The ARI score varied between 0.37 and 0.52. Also, based on Wilcoxon rank sum test we found no significant difference (*p* > 0.05) between the ARI scores of the TBI and HC for 3 of the 4 ROI-pairs; significant difference (*p* = 0.04) was only observed for the ROI-pair DMN-frontal-right and ECN-frontal-left. However, based on *t*-test no significant (*p* > 0.05) difference was found for all the 4 ROI-pairs.

**Table 2B:** **Sub-cluster-pair overlap analysis based on Adjusted rand index**.

**ROI-Pair**	**TBI mean(stdev)**	**Control mean(stdev)**
DMN frontal-left to ECN frontal-left	0.43 (0.12)	0.52 (0.12)
DMN frontal-right to ECN frontal-left	0.37 (0.17)	0.43 (0.08)
DMN frontal-left to ECN frontal-right	0.49 (0.19)	0.45 (0.18)
DMN frontal-right to ECN frontal-right	0.47 (0.11)	0.50 (0.24)

#### Voxel-pair consistency analysis

In this section we examine how consistently a voxel-pair is assigned to a significantly plastic sub-regional-pair across runs. In an ideal scenario, the EC approach will always (across multiple runs) identify a particular voxel-pair as either (a) existing in a significantly plastic sub-regional-pair or (b) not existing in a significantly plastic sub-regional-pair. To estimate the voxel-pair consistency we performed 5 independent runs per subject per ROI-pair. The results are summarized in Table 3; we found the voxel-pair consistency to be around 85% across all runs.

**Table 3:** **Link percentage consistency analysis**.

**ROI-Pair**	**TBI mean(stdev)**	**Control mean(stdev)**
DMN frontal-left to ECN frontal-left	85.43% (2.86)	87.98% (2.77)
DMN frontal-right to ECN frontal-left	83.18% (3.93)	85.47% (2.37)
DMN frontal-left to ECN frontal-right	86.63% (4.54)	85.57 (4.68)
DMN frontal-right to ECN frontal-right	86.15% (3.00)	86.92% (5.57)

#### Correlation analysis of plasticity estimates

If we add the number of connections contributing to positive plasticity and the number of voxel-wise connections contributing to negative plasticity, then we obtain overall plasticity; i.e., edges gained/lost (*gl*_**1**_).

In Section Simple Voxel-Level Approach we conducted a simple voxel-wise analysis by counting the number of voxel-wise functional connections that existed between a pair of ROI at session-1 and session-2. Thus, using the simple voxel-wise approach of section-Simple Voxel-Level Approach, we can derive another estimate for connections gained/lost (*gl*_**2**_). Even though the plasticity estimate *gl*_**2**_ is quite unrefined and may include functional connections that are not part of any statistically significant (see section Objective Function) sub-regional-pairs, it is reasonable to expect that the estimates *gl*_**1**_and *gl*_**2**_ should at least indicate similar plasticity effect in the brain. In order to examine this, for each subject for each ROI-pair we established the values: *gl*_**1**_ and *gl*_*2*_. Then we performed a simple correlation analysis. As we expected the correlation value was very high (between 0.97 and 1; see Figure S1). This helped us to further validate that the results obtained using evolutionary computation approach are reasonable.

## Discussion

In this paper we introduced a novel voxel-level procedure based on evolutionary computation (EC) to examine plasticity between an investigator-defined ROI-pair. In addition to allowing the researcher to define the ROI-pair *a priori*, the procedure is to our knowledge unique in that it automatically defines the correct spatial scales at which the plasticity is occurring. The procedure reliably detects statistically significant positively and negatively plastic sub-regional-pairs by simultaneously analyzing subject-specific BOLD-fMRI data collected from two fMRI-scan sessions that are separated by a finite duration of time.

Compared to averaging based method of examining functional connectivity, we found that the EC-based procedure showed improved sensitivity to subtle changes in connectivity occurring over time which otherwise would be hard to detect (see Figure 7 and Tables S1–S4). The subject specific plasticity estimates (i.e., % voxel-wise connections contributing to positive and negative plasticity) obtained using the EC approach across multiple independent runs were highly consistent (see section Examining Functional Plasticity); the standard deviation of negative and positive plasticity estimates were less than 0.5% for almost all cases (and Tables S1–S4).

Even though the EC approach was developed to estimate plasticity, in the future these sub-regional-pairs may serve as functionally discrete regions for further analysis (i.e., plasticity-specific brain parcellation for network analysis). For such applications, it would be important that the sub-regional-pairs overlap significantly across independent runs. We used two measures to quantify the reliability of the EC approach in this secondary sense. First, we documented the spatial overlap between the sub-regional-pairs found across independent runs using the Sorenson-dice index (a score of 0 means no overlap and 1 means complete overlap). Second, we documented the extent to which the mappings of voxel-pairs to sub-regional-pairs are consistent across independent runs according to the ARI score (a score of 0 means minimal overlap due to chance and 1 means complete overlap). On average, the Sorenson overlap score was 0.8 and the ARI score was between 0.37 and 0.52, which are comparable to the spatial overlap across multiple runs obtained using other voxel-based approaches for brain parcellation (Thirion et al., 2014; Wang et al., 2015).

Since the overlap score is quite high (Sorenson score = 0.8), the solutions are very similar in terms of the spatial location they encode, and therefore, the sub-regional-pairs detected may be reliably used for further *post hoc* analysis. As one more step to document the overlap between the solutions found by EC across multiple runs, we analyzed the consistency by which voxel-pairs are detected as a part of a significantly plastic sub-regional-pair. We found that the voxel-pair consistency to be highly reliable (85%). This consistency is particularly impressive given that most voxelwise clustering approaches are designed primarily for data parcellation (clustering of homogeneous voxels) and do not consider pairs of clusters and do not utilize multiple fMRI-scan session data simultaneously, which are added dimensions of complexity in the current approach. Overall, the EC approach showed good consistency in terms of plasticity estimates and also spatial overlap across independent runs. It was a primary goal of this work to validate this EC-approach, so we did not include additional constraints on the algorithm, but we anticipate the spatial-overlap for identification of sub-regional pairs can be further improved by imposing physiologically or anatomically relevant constraints during the evolutionary search.

Using this EC procedure we can quantify plasticity on the population level, including the typical levels of positive and negative plasticity in the HC brain (see Figure 7). This information will provide a reference point for examining the positive/negative plasticity during any developmental process (e.g., learning, aging, recovery). For example, based upon this HC baseline variability, when examining the TBI cases, we see that 11/14 TBI subjects show heightened positive plasticity, and about 2/14 TBI subjects (subject-1 and subject-5) show heightened negative plasticity between DMN-frontal right and ECN-frontal right. These findings could be very important. A sub-regional-pair showing significant positive functional plasticity indicates that across time the two sub-regions have become more synchronized, and therefore, there is a possibility that the amount of metabolic energy consumed by the two regions to maintain synchronization may have increased as well (Nugent et al., 2015). In context of the current work, the positive plasticity could imply a heightened metabolism in the brain of a TBI associated with elevated synchronization between DMN and ECN. By contrast, negative plasticity after TBI has been linked to disruption in specific white matter tracts connecting the regions (Bonnelle et al., 2011; Fagerholm et al., 2015). The results found using the EC based approach is quite consistent with what has been demonstrated in the literature regarding the plasticity between DMN and ECN during recovery from TBI. The hyperconnectivity reported here has been observed in this and other datasets of individuals with TBI revealing increased synchrony in large-scale networks (Nakamura et al., 2009; Sharp et al., 2011; Hillary et al., 2011a,b; Bonnelle et al., 2012; Caeyenberghs et al., 2012).

There are several advantages afforded by the EC-approach used here. First, it offers previously unavailable sensitivity to the sub-regional response within an investigator defined ROI-pair. That is, the current approach permits the investigator to select theoretically relevant ROIs for hypothesis testing while maintaining sensitivity to the change in the functional relationship between the ROIs occurring at a much finer scale than the ROIs selected. As a secondary characteristic of this approach, the investigator is afforded the unique opportunity to examine functionally discrete subdivisions within the ROI-pair, which may be helpful to examine subtle functional responses between brain regions. For example, as shown in Figure 8, between DMN-frontal left and ECN-frontal right, we found several significantly plastic sub-regional-pairs which would have remained undetected by conventional averaging based approach. In the context of TBI literature, this approach may be very useful in examining the relationship of several coarsely defined hub regions to other sub-networks in the brain. For example, hub regions such as the PCC thought to be central to the DMN functioning is now also recognized to hold differential responses to goal-directed and internal-state networks depending upon where within the PCC the investigator is sampling data (Leech et al., 2011). Based on the results discussed in this article, we anticipate the EC based procedure to be well suited for such research problems where the investigator defined ROIs are coarsely defined and may contain within ROI heterogeneity.

Finally, a very important feature of the EC approach is that it uses two fMRI-scan session data simultaneously, thereby, allowing the sub-regional-pairs to be held constant across time. Simultaneously using multiple fMRI-scan session data is an important issue that is under-represented in the imaging literature. For any investigation of brain plasticity, if the regions are not held constant across time, then interpreting the results can become very challenging. The EC based procedure thus contributes to this important research problem.

It should be noted that, consistent with traditional pre-processing steps, we used a 6 mm smoothing kernel (2 voxels wide). While some degree of spatial smoothing is standard for maximizing signal-to-noise-ratio in fMRI datasets and helps to minimize ringing artifacts (Lindquist and Wager, 2008), spatial blurring can introduce spurious local connectivity. However, because we use an identical kernel for all analyses (e.g., averaging vs. EC comparison) and the physical distances between the ROIs chosen in these analyses are greater than the smoothing kernel, we do not anticipate a significant effect of smoothing on the primary findings. For future applications, if the EC approach is used to examine within-ROI plasticity (instead of between-ROI plasticity) then a large smoothing kernel could influence the local connectivity results. EC is a flexible paradigm and we hope to integrate adaptive smoothing (Lindquist and Wager, 2008) to this procedure for effective within-ROI plasticity analysis in the future.

In summary, we present a sensitive and reliable EC approach which has immediate importance for multiple areas of work in the cognitive and clinical neurosciences. We demonstrate sensitivity to the effects that would remain undetected using conventional approaches to examining between-ROI connectivity changes across time. The procedure will be very useful for any longitudinal design and, given recent emphasis on “precision medicine,” ideal for examining subtle connectivity changes over time in an individual while maintaining superior granularity with respect to the spatial dimensions of this change. The approach offers sensitivity to subtle between-ROI changes occurring during developmental, interventional, or clinical recovery processes, which may be very important for developing bio-markers for clinical applications. In the future, we hope to extend this work by integrating anatomically relevant information to constrain the evolutionary search and extend the EC framework to examine within-ROI plasticity.

## Author contributions

AR, CC, FH developed the methodology. AR, CC, FH designed the experiments and analysis. CC provided conceptual advice on computational aspect of this work. FH and RB provided conceptual advice on clinical utility of this work. AR developed the software programs and conducted the computational experiments. FH and RB collected the Traumatic brain injury data. AR and RB preprocessed the fMRI data. AR, CC, FH, and RB wrote the manuscript.

## Funding

This project was supported by the Social Sciences Research Institute, in University Park, PA and the Penn State CTSI Grant (UL Tr000127) from the National Center for Advancing Translational Sciences, National Institutes of Health. The content is solely the responsibility of the authors and does not necessarily represent the official views of the NIH.

### Conflict of interest statement

The authors declare that the research was conducted in the absence of any commercial or financial relationships that could be construed as a potential conflict of interest.
