# Supplementary material for: An Evolutionary Computation Approach to Examine Functional Brain Plasticity
Source: Front Neurosci. 2016 Apr 5;10:146. doi: 10.3389/fnins.2016.00146 (PMC4820463; doi:10.3389/fnins.2016.00146)
Supplement: Supplementary file 1 [file DataSheet1.ZIP › supplementary/supplementary_table_and_figure_captions.docx]

**Figure S1:** The linear relationship between the variables gl1 and gl2 for TBIs (row 1) and HCs (row 2) for all 4 ROI pairs; the correlation values are shown on the top of each plot. The columns represent the ROI-pairs: DMN-frontal left to ECN-frontal left, DMN-frontal right to ECN-frontal left, DMN-frontal left to ECN-frontal right, and DMN-frontal right to ECN-frontal right (from left to right).

**Table 1. Subject demographic information**

|  | **Age** | **Education** | **Gender** | **GCS** | **Months post**  **injury scan 1** | **Months post**  **injury scan 2** |
| --- | --- | --- | --- | --- | --- | --- |
| **TBI mean (std)** | 26.07 (6.56) | 13.42 (2.43) | 7 M; 7 F | 7.92 (4.96) | 3.36 (0.74) | 6.64 (1.45) |
| **HC mean (std)** | 36.42 (15.25) | 13.42 (1.88) | 7 M; 5 F | N/A | N/A | N/A |

**Table 2a. Sub-cluster-pair overlap analysis based on Sorenson score.**

| **ROI-Pair** | **TBI** mean(stdev) | **Control** mean(stdev) |
| --- | --- | --- |
| DMN frontal-left to ECN frontal-left | 0.79 (0.02) | 0.80 (0.03) |
| DMN frontal-right to ECN frontal-left | 0.80 (0.02) | 0.80 (0.02) |
| DMN frontal-left to ECN frontal-right | 0.80 (0.02) | 0.79 (0.02) |
| DMN frontal-right to ECN frontal-right | 0.82 (0.02) | 0.82 (0.01) |

**Table 2b. Sub-cluster-pair overlap analysis based on Adjusted rand index.**

| **ROI-Pair** | **TBI** mean(stdev) | **Control** mean(stdev) |
| --- | --- | --- |
| DMN frontal-left to ECN frontal-left | 0.43 (0.12) | 0.52 (0.12) |
| DMN frontal-right to ECN frontal-left | 0.37 (0.17) | 0.43 (0.08) |
| DMN frontal-left to ECN frontal-right | 0.49 (0.19) | 0.45 (0.18) |
| DMN frontal-right to ECN frontal-right | 0.47 (0.11) | 0.50 (0.24) |

**Table 3. Link percentage consistency analysis**

| **ROI-Pair** | **TBI** mean(stdev) | **Control** mean(stdev) |
| --- | --- | --- |
| DMN frontal-left to ECN frontal-left | 85.43% (2.86) | 87.98% (2.77) |
| DMN frontal-right to ECN frontal-left | 83.18% (3.93) | 85.47% (2.37) |
| DMN frontal-left to ECN frontal-right | 86.63% (4.54) | 85.57 (4.68) |
| DMN frontal-right to ECN frontal-right | 86.15% (3.00) | 86.92% (5.57) |

**Table S1:** % voxel-wise edges contributing to plasticity between DMN-frontal-left and ECN-frontal-left. The following values are established using 5 and 10 independent EC runs.

| **Subjects** | **% voxel-wise edges contributing**  **to positive plasticity mean**  **(stdev)**  **5 runs/10 runs** | **% voxel-wise edges contributing**  **to negative plasticity mean**  **(stdev)**  **5 runs/10 runs** |
| --- | --- | --- |
| TBI-1 | 7.69 (1.23)/7.67 (1.05) | -1.99 (0.33)/-1.94 (0.25) |
| TBI-2 | 6.33 (0.61)/6.54 (0.25) | -8.73 (0.51)/-8.70 (0.54) |
| TBI-3 | 8.21 (0.30)/8.05 (0.43) | -3.28 (0.37)/-3.27 (0.20) |
| TBI-4 | 5.93(0.18)/6.03 (0.28) | -2.15 (0.13)/-2.23 (0.21) |
| TBI-5 | 1.01 (0.22)/1.15 (0.19) | -9.03 (0.31)/-9.06 (0.34) |
| TBI-6 | 14.40 (0.67)/12.9 (0.98) | -1.33 (0.10)/-1.36 (0.33) |
| TBI-7 | 2.86 (0.16)/2.66 (0.30) | -4.60 (0.29)/-4.73 (0.21) |
| TBI-8 | 12.15 (0.74)/11.71 (1.61) | -0.34 (0.46)/-0.43 (0.46) |
| TBI-9 | 4.77 (0.48)/4.32 (0.31) | -3.47 (0.32)/-3.37 (0.33) |
| TBI-10 | 6.98 (0.24)/6.96 (0.4) | -6.07 (0.41)/-5.94 (0.50) |
| TBI-11 | 9.43 (0.65)/9.63 (0.83) | -1.58 (0.21)/-1.52 (0.50) |
| TBI-12 | 4.72 (0.25)/4.65 (0.15) | -3.65 (0.26)/-3.61 (0.31) |
| TBI-13 | 3.28 (0.38)/2.99 (0.33) | -7.87 (0.34)/-8.03 (0.43) |
| TBI-14 | 4.21(0.29)/4.13 (0.57) | -0.62 (0.71)/-1.47 (0.32) |
| HC-1 | 0.76 (0.10)/0.82 (0.14) | -3.11 (0.03)/-3.13 (0.05) |
| HC-2 | 6.56 (0.21)/6.46 (0.22) | -2.19 (0.14)/-2.06 (0.17) |
| HC-3 | 2.48 (0.25)/2.55 (0.20) | -2.96 (0.12)/-2.78 (0.16) |
| HC-4 | 7.26 (0.16)/7.26 (0.24) | -3.28 (0.15)/-3.33 (0.27) |
| HC-5 | 6.65 (0.29)/6.80 (0.46) | -0.79 (0.07)/-0.86 (0.35) |
| HC-6 | 1.07 (0.08)/1.09 (0.20) | -5.49 (0.15)/-5.42 (0.12) |
| HC-7 | 1.65 (0.43)/1.90 (0.36) | -4.00 (0.28)/-3.97 (0.29) |
| HC-8 | 4.47 (0.53)/4.60 (0.42) | -1.47 (0.05)/-1.63 (0.15) |
| HC-9 | 1.26 (0.21)/1.40 (0.17) | -3.32 (0.17)/-3.25 (0.24) |
| HC-10 | 0.82 (0.18)/0.92 (0.09) | -5.16 (0.20)/-5.13 (0.23) |
| HC-11 | 1.68(0.37)/1.72 (0.31) | -5.52 (0.15)/-5.66 (0.25) |
| HC-12 | 10.12 (0.84)/9.84 (0.81) | -0.09 (0.18)/-0.04 (0.10) |

**Table S2:** % voxel-wise edges contributing to plasticity between DMN-frontal-left and ECN-frontal-right. The following values are established using 5 and 10 independent EC runs.

| **Subjects** | **% voxel-wise edges contributing**  **to positive plasticity mean**  **(stdev)**  **5 runs/10 runs** | **% voxel-wise edges contributing**  **to negative plasticity mean**  **(stdev)**  **5 runs/10 runs** |
| --- | --- | --- |
| TBI-1 | 3.54 (0.19)/3.44 (0.21) | -7.12 (0.30)/-7.21 (0.30) |
| TBI-2 | 5.07 (0.29)/5.29 (0.56) | -5.86 (0.02)/-5.77 (0.15) |
| TBI-3 | 3.39 (0.16)/3.26 (0.16) | -5.29 (0.17)/-5.19 (0.36) |
| TBI-4 | 2.15 (0.31)/2.06 (0.21) | -2.72 (0.25)/-2.72 (0.14) |
| TBI-5 | 0.35 (0.13)/0.45 (0.07) | -12.54 (0.31)/-12.45 (0.19) |
| TBI-6 | 11.11 (0.74)/10.79 (0.81) | -2.96 (0.57)/-3.17 (0.28) |
| TBI-7 | 0.22 (0.15)/0.31 (0.17) | -9.74 (0.22)/-9.72 (0.29) |
| TBI-8 | 3.04 (0.70)/2.87 (0.61) | -3.02 (0.23)/-2.78 (0.29) |
| TBI-9 | 4.28 (0.19)/4.12 (0.35) | -2.97 (0.19)/-2.95 (0.34) |
| TBI-10 | 10.25 (0.93)/10.00 (0.95) | -0.70 (0.21)/-0.71 (0.18) |
| TBI-11 | 2.85 (0.16)/2.95 (0.38) | -4.46 (0.37)/-4.4 (0.32) |
| TBI-12 | 3.48 (0.23)/3.23 (0.37) | -1.77 (0.21)/-1.63 (0.13) |
| TBI-13 | 3.73 (0.33)/4.09 (0.36) | -5.12 (0.36)/-4.95 (0.24) |
| TBI-14 | 3.79 (0.37)/3.50 (0.18) | -1.56 (0.26)/-1.75 (0.20) |
| HC-1 | 2.85 (0.26)/2.82 (0.19) | -0.36 (0.18)/-0.48 (0.19) |
| HC-2 | 15.64 (0.07)/15.69 (0.09) | -1.23 (0.07)/-1.19 (0.11) |
| HC-3 | 2.24 (0.35)/2.19 (0.28) | -2.00 (0.05)/-1.96 (0.15) |
| HC-4 | 6.74 (0.14)/6.88 (0.53) | -1.57 (0.25)/-1.68 (0.14) |
| HC-5 | 1.60 (0.20)/1.55 (0.19) | -2.70 (0.07)/-2.75 (0.09) |
| HC-6 | 1.66 (0.36)/1.70 (0.35) | -3.14 (0.21)/-3.15 (0.15) |
| HC-7 | 1.44 (0.15)/1.56 (0.29) | -3.35 (0.27)/-3.41 (0.24) |
| HC-8 | 1.64 (0.36)/1.69 (0.29) | -4.27(0.11)/-4.30 (0.19) |
| HC-9 | 0.57 (0.22)/0.94 (0.34) | -2.38 (0.14)/-2.42 (0.32) |
| HC-10 | 1.48 (0.31)/1.45 (0.30) | -4.40 (0.20)/-4.31 (0.21) |
| HC-11 | 2.00 (0.10)/1.91 (0.19) | -4.63 (0.18)/-4.69 (0.12) |
| HC-12 | 4.51 (0.17)/4.52 (0.30) | -0.99 (0.26)/-0.98 (0.18) |

**Table S3:** % voxel-wise edges contributing to plasticity between DMN-frontal-right and ECN-frontal-left. The following values are established using 5 and 10 independent EC runs.

| **Subjects** | **% voxel-wise edges contributing**  **to positive plasticity mean**  **(stdev)**  **5 runs/10 runs** | **% voxel-wise edges contributing**  **to negative plasticity mean**  **(stdev)**  **5 runs/10 runs** |
| --- | --- | --- |
| TBI-1 | 5.11 (0.42)/5.21 (0.33) | -2.85 (0.11)/-2.84 (0.16) |
| TBI-2 | 3.37 (0.75)/3.39 (0.56) | -5.03 (0.13)/-4.96 (0.18) |
| TBI-3 | 4.11 (0.37)/4.11 (0.22) | -1.83 (0.45)/-1.73 (0.33) |
| TBI-4 | 1.33 (0.43)/1.37 (0.34) | -2.88 (0.26)/-2.89 (0.25) |
| TBI-5 | 0.83 (0.18)/0.98 (0.19) | -10.28 (0.20)/-10.21 (0.16) |
| TBI-6 | 10.64 (0.90)/11.55 (0.87) | -1.76 (0.39)/-1.74 (0.46) |
| TBI-7 | 2.06 (0.25)/2.04 (0.13) | -3.68 (0.15)/-3.68 (0.22) |
| TBI-8 | 3.44 (0.70)/3.55 (0.50) | -1.32 (0.20)/-1.33 (0.15) |
| TBI-9 | 3.58 (0.26)/3.51 (0.25) | -2.37 (0.13)/-2.59 (0.13) |
| TBI-10 | 8.31 (1.53)/8.41 (1.12) | -2.53 (0.30)/-2.56 (0.29) |
| TBI-11 | 6.02 (0.74)/6.45 (0.49) | -1.51 (0.17)/-1.44 (0.34) |
| TBI-12 | 2.78 (0.31)/2.63 (0.26) | -1.43 (0.15)/-1.51 (0.24) |
| TBI-13 | 3.88 (0.23)/3.71 (0.30) | -4.96 (0.32)/-5.07 (0.31) |
| TBI-14 | 2.27 (0.28)/2.25 (0.27) | -3.16 (0.19)/-3.10 (0.12) |
| HC-1 | 2.09 (0.12)/2.06 (0.12) | -1.03 (0.10)-1.08 (0.12) |
| HC-2 | 2.45 (0.07)/2.54 (0.32) | -2.58 (0.24)/-2.72 (0.25) |
| HC-3 | 1.58 (0.19)/1.57 (0.17) | -1.89 (0.09)/-1.90 (0.09) |
| HC-4 | 3.55 (0.33)/3.72 (0.21) | -4.15 (0.11)/-4.02 (0.17) |
| HC-5 | 3.21 (0.37)/3.16 (0.44) | -0.88 (0.23)/-0.85 (0.15) |
| HC-6 | 2.51 (0.31)/2.42 (0.26) | -1.81 (0.09)/-1.86 (0.16) |
| HC-7 | 1.17 (0.14)/1.17 (0.14) | -3.62 (0.15)/-3.50 (0.12) |
| HC-8 | 2.66 (0.31)/2.74 (0.21) | -2.55 (0.25)/-2.36 (0.23) |
| HC-9 | 1.57 (0.12)/1.57 (0.18) | -1.45 (0.13)/-1.46 (0.24) |
| HC-10 | 1.15 (0.25)/0.92 (0.13) | -3.48 (0.17)/-3.56 (0.10) |
| HC-11 | 1.52 (0.17)/1.66 (0.16) | -4.04 (0.35)/-4.02 (0.24) |
| HC-12 | 4.54 (0.20)/4.46 (0.18) | -0.97 (0.06)/-0.98 (0.07) |

**Table S4:** % voxel-wise edges contributing to plasticity between DMN-frontal-right and ECN-frontal-right. The following values are established using 5 and 10 independent EC runs.

| **Subjects** | **% voxel-wise edges contributing**  **to positive plasticity mean**  **(stdev)**  **5 runs/10 runs** | **% voxel-wise edges contributing**  **to negative plasticity mean**  **(stdev)**  **5 runs/10 runs** |
| --- | --- | --- |
| TBI-1 | 2.25 (0.32)/2.24 (0.39) | -10.78 (0.33)/-10.59 (0.35) |
| TBI-2 | 9.50 (0.18)/9.91 (0.53) | -3.38 (0.28)/-2.96 (0.42) |
| TBI-3 | 2.59 (0.22)/2.70 (0.42) | -6.90 (0.12)/-7.09 (0.41) |
| TBI-4 | 4.88 (0.43)/5.02 (0.11) | -3.09 (0.22)/-3.03 (0.23) |
| TBI-5 | 0.97 (0.22)/0.92 (0.20) | -10.03 (0.10)/-10.09 (0.14) |
| TBI-6 | 10.09 (1.36)/10.35 (1.36) | -2.73 (0.25)/-2.90 (0.33) |
| TBI-7 | 4.90 (0.23)/4.47 (0.56) | -5.95 (0.25)/-5.89 (0.31) |
| TBI-8 | 7.26 (0.70)/7.37 (0.52) | -2.56 (0.39)/-2.31 (0.38) |
| TBI-9 | 13.25 (1.26)/12.88 (1.06) | -0.68 (0.30)/-0.90 (0.31) |
| TBI-10 | 6.32 (1.04)/6.37 (0.23) | -6.14 (0.17)/-5.94 (0.25) |
| TBI-11 | 9.65 (0.89)/9.27 (1.24) | -2.32 (0.38)/-2.53 (0.36) |
| TBI-12 | 5.06 (0.46)/4.88 (0.31) | -2.24 (0.32)/-2.30 (0.23) |
| TBI-13 | 14.75 (1.08)/13.38 (1.82) | -3.25 (0.66)/-3.10 (0.34) |
| TBI-14 | 3.59 (0.34)/3.76 (0.36) | -2.93 (0.39)/-2.96 (0.17) |
| HC-1 | 1.92 (0.14)/1.96 (0.23) | -1.49 (0.11)/-1.48 (0.10) |
| HC-2 | 8.59 (0.34)/8.54 (0.15) | -1.71 (0.17)/-1.72 (0.08) |
| HC-3 | 2.37 (0.35)/2.47 (0.25) | -2.73 (0.29)/-2.68 (0.25) |
| HC-4 | 3.93 (0.39)/3.81 (0.25) | -5.95 (0.21)/-6.04 (0.16) |
| HC-5 | 0.98 (0.14)/0.94 (0.14) | -4.45 (0.26)/-4.56 (0.16) |
| HC-6 | 1.88 (0.25)/1.66 (0.23) | -4.05 (0.19)/-3.86 (0.19) |
| HC-7 | 1.69 (0.04)/1.60 (0.17) | -6.46 (0.07)/-6.51 (0.12) |
| HC-8 | 4.09 (0.45)/4.18 (0.32) | -3.99 (0.29)/-4.08 (0.34) |
| HC-9 | 2.77 (0.22)/2.89 (0.19) | -0.95 (0.31)/-0.94 (0.09) |
| HC-10 | 0.12 (0.03)/0.06 (0.06) | -8.76 (0.00)/-8.81 (0.07) |
| HC-11 | 1.05 (0.11)/1.20 (0.23) | -5.19 (0.32)/-5.14 (0.29) |
| HC-12 | 5.41 (0.24)/5.21 (0.29) | -1.52 (0.26)/-1.48 (0.25) |

**Table S5:** The brain regions that are part of DMN-frontal-right/left and ECN-frontal-right/left

| **ROI-ID** | **AAL region name** |
| --- | --- |
| DMN-frontal-left | Left superior frontal gyrus (medial part),  Left superior frontal gyrus (medial orbital part),  Left gyrus rectus |
| DMN-frontal-right | Right superior frontal gyrus (medial part),  Right superior frontal gyrus (medial orbital part),  Right gyrus rectus |
| ECN-frontal-left | Left superior frontal gyrus (dorsolateral),  Left middle frontal gyrus (lateral part),  Left opercular part of inferior frontal gyrus,  Left area triangularis,  Left orbital part of inferior frontal gyrus |
| ECN-frontal-right | Right superior frontal gyrus (dorsolateral),  Right middle frontal gyrus (lateral part),  Right opercular part of inferior frontal gyrus,  Right area triangularis,  Right orbital part of inferior frontal gyrus |

**Table S6:** Summary of runtime of the evolutionary computation based procedure

| **ROI-pair** | **Runtime mean(stdev) in minutes** |
| --- | --- |
| DMN-frontal-left/ECN-frontal-left | 15.96 (5.31) |
| DMN-frontal-left/ECN-frontal-right | 15.41 (5.38) |
| DMN-frontal-right/ECN-frontal-left | 16.04 (4.29) |
| DMN-frontal-right/ECN-frontal-right | 17.25 (4.76) |
