# Supplementary figures and images for: An Evolutionary Computation Approach to Examine Functional Brain Plasticity
Source: Front Neurosci. 2016 Apr 5;10:146. doi: 10.3389/fnins.2016.00146 (PMC4820463; doi:10.3389/fnins.2016.00146)

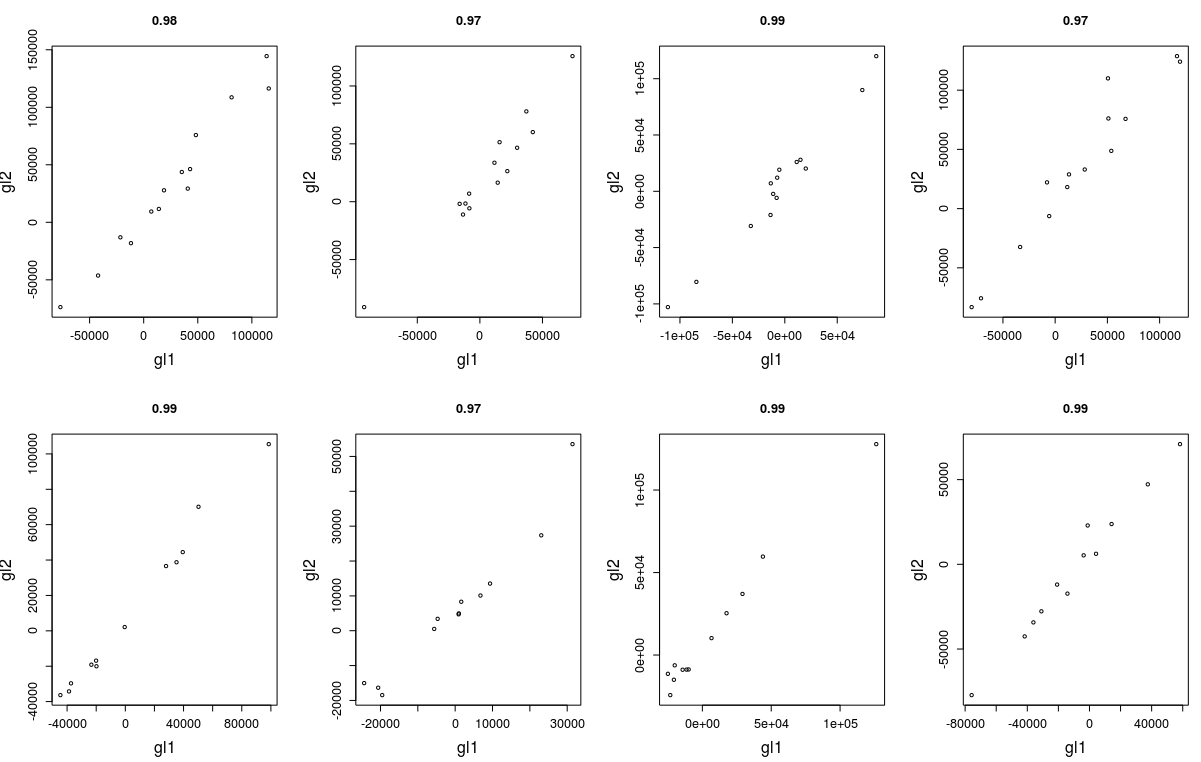

Supplement: Supplementary file 1 [file DataSheet1.ZIP › supplementary/Figure_S1.tiff]
